# Soluble starch synthase IVa determines cooked rice elongation in waxy and non-waxy rices (*Oryza sativa* L.)

**DOI:** 10.3389/fpls.2026.1922515

**Published:** 2026-09-11

**Authors:** Ekawat Chaichoompu, Siriphat Ruengphayak, Burin Thunnom, Wanchana Aesomnuk, Watchareewan Jamboonsri, Samart Wanchana, Wintai Kamolsukyeunyong, Siwaret Arikit, Theerayut Toojinda, Apichart Vanavichit

**Affiliations:** 1 Interdisciplinary Graduate Program in Genetic Engineering and Bioinformatics, Kasetsart University, Chatuchak, Bangkok, Thailand; 2 Rice Science Center, Kasetsart University, Kamphaeng Saen, Nakhon Pathom, Thailand; 3 Innovative Plant Biotechnology and Precision Agriculture Research Team, National Center for Genetic Engineering and Biotechnology (BIOTEC), National Science and Technology Development Agency (NSTDA), Khlong Nueng, Khlong Luang, Pathum Thani, Thailand; 4 Department of Agronomy, Faculty of Agriculture at Kamphaeng Saen, Kasetsart University, Nakhon Pathom, Thailand

**Keywords:** cooked rice elongation, haplotype, marker-assisted selection, non-waxy rice, pigmented rice, QTL-seq, soluble starch synthase IVa (SSIVa), waxy rice

## Abstract

**Significance:**

Cooked rice elongation (CRE) is an attractive grain quality reported only in Basmati from India and Pakistan and in Paw San Hmwe from Myanmar. Several reports on the discovery of genes controlling CRE were unequivocal in rice. Here, we design new experiments to uncover genes that significantly affect CRE in waxy and non-waxy rice varieties.

**Materials and methods:**

We created 105 BC_1_F_2_ lines derived from a cross between a high-CRE waxy rice, NHN, and Pink+4 (#20A09), a low-CRE, low-glycemic-index, non-waxy rice, to generate two distinct phenotypic pools for QTL-seq analysis: high-CRE and low-CRE. 31 waxy, non-waxy, and pigmented rice varieties, including a few Basmati accessions, were the test panel for the identified single-nucleotide polymorphic markers (SNPs) developed from the candidate gene.

**Results:**

Six significant QTLs, localized by ΔSNP Indexes, were mapped on chromosomes 1 (*qCRE1*), 4 (*qCRE4*), 7 (*qCRE7*), 10 (*qCRE10.1, qCRE10.2*) and 12 (*qCRE12*). Significantly, the *qCRE1*, which spans 29.3-30.2 Mb, showed the strongest ΔSNP Index with a significant phenotypic variance explained (PVE), whereas *qCRE4, qCRE7, qCRE10.1, qCRE10.2* and *qCRE12* were non-significant. Specifically, SNP-*SSIVa*_*1_30038156* (R01030038156), a non-synonymous mutation encoding the A-to-G variant in exon 5 that strongly discriminated between low and high-CRE in waxy, is co-localised in the exon 5 of the *Soluble Starch Synthase IVa* (*SSIVa*) on *qCRE1*. In this critical region, data mining identified 14 informative SNPs in the coding and non-coding sequence of the *SSIVa* gene. Moreover, four haplotypes (HAP) derived from seven informative SNPs in *SSIVa* correctly classified the rice accessions into the extra-CRE (HAP1), high-CRE (HAP2), and low-CRE (HAP 3 and 4). All extra-CRE rice varieties, including 11 Basmati and Paw San Hmwe, were classified in HAP1. While high-CRE rice varieties, including NHN and six rice varieties, were designated in HAP2. 13 low-CRE rice varieties were classified in either HAP3 or HAP4.

**Conclusion:**

For the first time, this finding signifies the genetic impact of *SSIVa* on CRE in both waxy and non-waxy rice varieties. The corresponding haplotypic DNA markers will benefit rice breeders aiming to improve elite rice varieties with attractive cooked-rice elongation through marker-assisted selection.

## 1 Introduction

Rice is not only one of the most consumed caloric foods but also an important source of income for farmers in the tropics. Rice grain quality is a complex trait comprising physical and biochemical properties, nutritional profile, fragrance, and cooked-rice characteristics. Cooked rice elongation (CRE) is a unique physical property, commonly observed in Basmati rice from India and Pakistan, as well as in Myanmar’s Paw San Hmwe (Oo et al., 2015). CRE is significant because it is widely used as a marker of cooking quality, consumer preference, and breeding value, especially in premium and Basmati-type rice. CRE is an important or even defining trait of high-quality rice, with consumers generally preferring grains that elongate lengthwise rather than swell in width because it gives cooked rice a finer appearance (Golam and Prodhan, 2013; Arikit et al., 2019; Rajendran et al., 2021; Marasingha et al., 2024). Pusa Basmati 1121, a variety with an exceptionally high cooked kernel elongation ratio of 2.5 and cooked kernel length up to 22 mm, is reported to have set new standards in the Basmati market and generated major export value and farmer returns (Singh et al., 2018; Rajendran et al., 2021; Haider, 2017; Sanusi et al., 2022).

In non-waxy rice, numerous quantitative trait loci (QTLs) associated with CRE are located near genes involved in starch biosynthesis, including major loci near starch biosynthesis genes like *Granule-bound Starch Synthase I* (*GBSSI*), *Soluble Starch Synthase IIa* (*SSIIa*), *Starch Branching Enzyme IIb* (*SBEIIb*), and *Grain Size 3* (*GS3*), as well as novel regions across multiple chromosomes (Qiu et al., 2021; Arikit et al., 2019; Zhang et al., 2004; Ge et al., 2005; Malik et al., 2022; Yang et al., 2013; Ahn et al., 1993; Govindaraj et al., 2009; Tian et al., 2005; Ramkumar et al., 2010; Wang et al., 2007; Thi et al., 2020; Amarawathi et al., 2008; Fujita et al., 2022; Johar and Salgotra, 2025). Studies using genome-wide association studies (GWAS), QTL mapping, and bulked segregant analysis have revealed that CRE is polygenic, with both additive and non-additive gene actions, and significant genotype-by-environment interactions (Jin et al., 2010; Halim et al., 2021; Shen et al., 2005; Kumar et al., 2020; Faruq et al., 2004; Saidon et al., 2020; Irshad et al., 2021; Zheng et al., 2023). The trait is also closely linked to other grain quality attributes such as AC, GT, and grain length (Anjan et al., 2025; Roy et al., 2021; Shen et al., 2011; Misra et al., 2017).

Donor-specific QTLs of CRE are variable. There were reports on a major gene with modifiers in Basmati 370 (Faruq et al., 2004), Zhenshan 97 × Minghui 63 (Ge et al., 2005), Basmati 370 x *O. glaberrima* (Li et al., 2004), Zhenshan 97 x Delong (Wang et al., 2007), indica x japonica cross (Liu et al., 2008), Basmati x PTT1 (Arikit et al., 2019). These QTLs have not been accurately pinpointed in the CRE’s functional markers for marker-assisted selection.

In general, CRE is influenced by amylose content (AC), amylopectin (AP), gelatinization temperature (GT), dietary fiber (DF), and protein content (PC), which also control the physical and biochemical properties of whole-grain rice (Bao et al., 2001; Arikit et al., 2019; Zhang et al., 2021; Zheng et al., 2023; Okpala et al., 2017; Ball and Morell, 2003; Myers et al., 2000). Five distinct families of *Starch Synthases* (*SS*) have been characterized in plants. Four families (*SSI–SSIV*) predominantly occur as soluble enzymes, while the fifth, *Granule-bound Starch Synthase* (*GBSS*), is associated with starch granules. Mutants for all *SS* families have been generated and analyzed across various plant and algal systems, except *SSIV*, for which limited insights exist. Recent studies by Hirose and Terao (2004) demonstrated that the two *SSIV* genes in rice (*SSIVa* and *SSIVb*) are ubiquitously expressed across the plant and maintain relatively consistent expression levels during grain filling. Nevertheless, direct genetic evidence for the role of *SSIV* in starch biosynthesis remains lacking. Variations in *SSIV* alleles have been shown to significantly influence rice quality, particularly concerning gelatinization and thermal properties of starch, even within the same genetic background defined by the major *Waxy* (*GBSSI*) genes. This finding suggests that *SSIV* may contribute to the elongation of starch chains (Xu et al., 2020). Furthermore, investigations into SSIV-deficient mutants in *Arabidopsis thaliana* have revealed a novel function for this enzyme in modulating starch granule formation (Roldán et al., 2007; Toyosawa et al., 2016). *SSIV* is expressed in both the leaves and seeds of wheat (Hawkins et al., 2021). Comparative analysis of the glycosyl transferase domains in wheat and rice SSIV reveals amino acid-level differences, which may indicate functional divergence between these species (Irshad et al., 2021). Therefore, the genetic mechanism of CRE is still unclear. Nonetheless, environmental factors such as temperature during grain filling and post-harvest ageing also play significant roles in CRE (Juliano and Perez, 1984; Golam and Prodhan, 2013; Halim et al., 2021; Irshad et al., 2021; Halim et al., 2023; Okpala et al., 2021).

Laos and Thailand are the only countries that consume glutinous rice as a primary stable food. In Thailand, glutinous rice is vital for household consumption in the north and northeast. Glutinous rice is mainly used to create traditional sweet dishes in Thailand. Nonetheless, waxy rice with high-CRE is uncommon yet more attractive, adding value to traditional Thai desserts, which are mostly cooked with coconut milk. We developed NHN, a rare new waxy rice variety that not only shows extra-long, slender grains and is highly aromatic, but also has CRE. We developed a BC_1_F_2_ population from the cross NHN × Pink+4 (#20A09), a high-amylose, low-glycemic-index, non-CRE rice variety, and identified three candidate QTLs, particularly the QTL on chromosome 1, which showed a significant ΔSNP index associated with the CRE in waxy rice. The strongest candidate gene for CRE was identified in the QTL region as *SSIVa*. Furthermore, haplotypes were identified from seven informative SNPs in the *SSIVa* coding sequence, thereby effectively grouping diverse waxy and non-waxy rice varieties into extra-high, high, and low-CRE categories.

## 2 Materials and methods

### 2.1 Parental materials and germplasm

The 31 rice varieties were provided by Rice Science Center, Kasetsart University, Thailand. NHN and Pink+4 (#20A09) were used as parents for crossing. NHN is an extra-long grain, aromatic, glutinous rice with high-GT and high-CRE, while Pink+4 (#20A09) is a long grain, aromatic, high-AC rice with low-GT and low-CRE. The milled-grain length is 8.44 ± 0.03 mm and 7.42 ± 0.03 mm for NHN and Pink+4 (#20A09), respectively. Following cooking, grain length increased to 14.47 ± 0.07 mm in NHN and 10.11 ± 0.02 mm in Pink+4 (#20A09). The CRE ratio was calculated as the ratio of cooked grain length to uncooked grain length. NHN exhibited a high-CRE ratio of 1.72±0.10, whereas Pink+4 (#20A09) showed a low-CRE ratio of 1.37±0.08.

### 2.2 Backcross mapping population

We developed a BC_1_F_2_ mapping population derived from backcrossing F_1_ of a cross between NHN and Pink+4 (#20A09) to NHN. Due to the wide incompatibility between NHN and Pink+4 (#20A09), only a limited number of BC_1_F_1_ lines were fertile and able to generate 242 BC_1_F_2_ plants. However, only 105 families could set BC_1_F_3_ seeds for the CRE phenotyping.

### 2.3 Phenotyping

Physical properties and CRE were analyzed for 105 BC_1_F_2_-derived F_3_ seeds. Five polished grains of each BC_1_F_3_ family were collected and evaluated for CRE by placing the grain in a 0.2 ml microtube, presoaked in distilled water for 30 min at ambient temperature and cooked in a 100°C water bath for 15 min. Grain lengths were measured individually before and after being cooked using Java image processing program (ImageJ V1.4). The CRE ratio was calculated as the ratio of cooked grain length to uncooked grain length.

### 2.4 QTL- seq analysis

QTL-seq analysis was designed to identify tightly linked DNA markers to the target QTL and to perform gene finding, utilizing whole-genome resequencing of DNA bulks from opposite sets of progeny (Giovannoni et al., 1991; Takagi et al., 2013; Zou et al., 2016). We performed QTL-seq analysis on extreme pools of selected BC_1_F_2_ lines, comprising 17 high-CRE and 15 low-CRE lines, derived from a backcross between NHN and Pink+4 (#20A09).

Genomic DNA Isolation: Genomic DNAs were extracted individually and pooled to form the high-CRE and low-CRE Pools, following the DNeasy Plant Mini Kit (QIAGEN, USA). DNA-seq libraries of high-CRE and low-CRE bulks, including the parents, were whole-genome sequenced using the Illumina HiSeq-2500 platform (Illumina, Inc., USA).

Genome sequencing: Genomic DNA from the two parents and two extreme bulks, high-CRE and low-CRE, was used for whole-genome sequencing on an Illumina HiSeq. 2500. Raw paired-end sequences generated 63 and 106 million reads for NHN and Pink+4 (#20A09), respectively. There were approximately 63 and 66 million reads of paired-end sequences for high-CRE bulk and low-CRE bulk, respectively. After trimming with a high-quality base containing Phred scores of 30 or greater, only 56, 80, 50 and 48 million reads were retained in NHN, Pink+4 (#20A09), low-CRE bulk and high-CRE bulk, respectively.

SNP index: The QTL-Seq was performed using the QTL-seq pipeline as described previously (Takagi et al., 2013). We used NHN as a reference for this analysis pipeline. For each SNP position, the SNP index was calculated from the differential frequency of SNPs in the high-CRE and low-CRE bulks. SNP Indexes <0.3 were removed in both pools, and the ΔSNP index was then calculated using the following formula:


ΔSNP index=SNP index (high−CRE bulk)−SNP index (low−CRE bulk)

The distribution of the moving-mean average of the SNP index and ΔSNP index was calculated for each 2 MB sliding window and plotted against the rice chromosomes. The critical genomic regions for CRE were identified from the sliding window plots.

### 2.5 SNP markers development

Kompetitive Allele-Specific PCR (KASP) markers were developed based on selected SNPs identified within the selected QTL regions. KASP genotyping assays were performed following the manufacturer’s protocol (LGC Genomics; http://www.lgcgenomics.com). Reactions were conducted in a 1536-well plate format using the Meridian robotic liquid dispensing system (LGC, Biosearch Technology), with a total reaction volume of 1 µL. Each reaction contained 1.5 µL of dried genomic DNA template (2–10 ng/µL), 0.015 µL of KASP assay mix (100 µM), and 0.5 µL of KASP master mix (2X).

PCR amplification was performed using a Hydrocycler water-bath thermal cycler (LGC, Biosearch Technology). The thermal cycling conditions consisted of an initial denaturation at 94 °C for 5 min, followed by 10 touchdown cycles of 94 °C for 20 s and 61 °C for 60 s, with the annealing temperature decreasing by 0.6 °C per cycle. This was followed by 27 cycles of 94 °C for 20 s, 55 °C for 30 s, and a final extension at 37 °C for 1 min.

Fluorescence signals of the amplified products were detected using a PHERAstar FS high-throughput microplate reader (BMG Labtech). Genotype calling was performed based on allele-specific fluorescence clustering.

### 2.6 Statistical analysis

Single-marker analysis was performed using linear regression implemented in R (http://www.r-project.org) with the linear model function. Genotypic data were derived from KASP marker assays, while phenotypic data were represented by the CRE ratio, calculated as the ratio of cooked grain length to uncooked grain length.

### 2.7 Haplotype identification

To validate the association of *SSIVa* and CRE, we searched a broader spectrum of waxy, non-waxy, and pigmented rice varieties in our in-house resequenced seed collection archive using the 14 SNP genotypes in *SSIVa* on chromosome 1 and the four SNP genotypes in *GBSSI* and *SSIIa* on chromosome 6. We identified 31 accessions with polymorphisms in the region. Eleven polymorphic SNPs were genotyped using KASP markers and phenotyped for the corresponding CRE.

## 3 Results

### 3.1 Hybrid sterility

**Figure 1:**
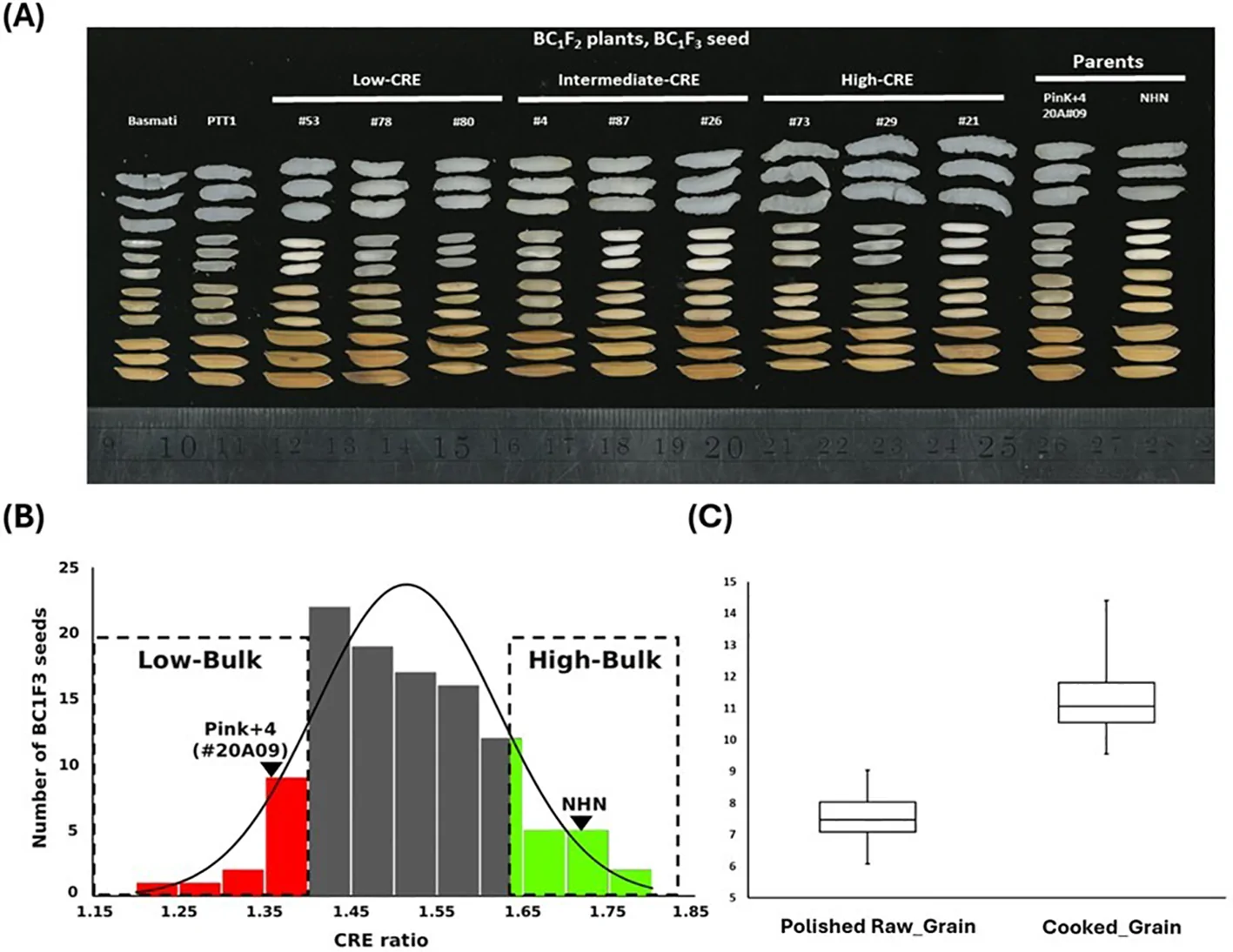
Cooked rice elongation ratio (CRE) of the parental lines, NHN and Pink+4 (#20A09), and the BC_1_F_2_-derived F_3_ seed population. **(A)** Comparison of CRE ratios among the parental lines, reference controls Basmati (high-CRE) and PTT1 (low-CRE), and representative BC_1_F_3_ seeds showing segregation for CRE. **(B)** Frequency distribution of CRE values in the BC_1_F_3_ and parental lines used for QTL-seq analysis. Shaded regions represent the upper and lower tails of the CRE distribution selected for DNA bulking and whole-genome sequencing. **(C)** Box plots of average raw grain length and cooked grain length among 105 BC_1_F_3_ including two parental lines.

The crosses between NHN and Pink+4 (#20A09) were partially infertile. We hypothesized that the two parents were genetically divergent, which can typically cause infertility in rice. From 242 BC_1_F_2_ lines, only 105 lines (43.38%) were fertile. Moreover, the infertility was also due to high daytime temperatures during the growing season (Matsui et al., 2001; Jagadish et al., 2007; Inthima et al., 2026). Nonetheless, the CRE distribution in the 105 BC_1_F_2_-derived F3 seed population was close to normal, ranging from 1.22 to 1.76, with a typical 3:1 heterozygous-to-homozygous ratio. The two opposite CRE groups, comprising 15 extremely low-CRE and 17 extremely high-CRE, clearly exhibited different lengthwise elongation in the low- and high-CRE BC_1_F_3_ seeds (**Figure 1B**; **Supplementary Figure 1**). The opaque appearance, commonly observed among waxy rice varieties, including NHN, confirmed the 3:1 ratio for the opaque-to-translucent grain in BC1F3 seeds (**Figures 1A, C**; **Supplementary Table 1**).

### 3.2 Physical characterization

We compared NHN to Pink+4 in terms of the physical dimensions of uncooked and cooked rice. The uncooked grain lengths (UG) of NHN and Pink+4 (#20A09) were 8.44 ± 0.03 mm and 7.42 ± 0.03 mm, respectively, or a 1.02 mm difference. The cooked grain lengths (CG) were elongated to 14.47 ± 0.07 mm and 10.14 ± 0.02 mm, respectively, at CRE ratios of 1.72 and 1.37. When comparing Basmati (high-CRE) and PTT1 (low-CRE), as the two control rice varieties, we found the grain length of milled/cooked rice was 14.14/7.64 and 10.44/7.33, respectively, or 1.85 and 1.42, respectively. Despite the smaller CRE for NHN compared to Basmati, the extra grain length of NHN resulted in longer cooked rice (**Figures 1A, B**; **Supplementary Table 1**).

### 3.3 Whole-genome sequencing and QTL-seq analysis

Whole-genome resequencing was performed on the parental lines, NHN and Pink+4 (#20A09), as well as the contrasting CRE bulks derived from the BC_1_F_2_ population. A total of 63.88 million and 106.09 million raw reads were generated for NHN and Pink+4 (#20A09), respectively, with average sequencing depths of approximately 19.81x and 25.65×. Sequencing of the bulked samples produced 66.23 million reads for the low-CRE bulk, and 63.06 million reads for the high-CRE bulk (**Supplementary Table 2**).

Low-quality reads and adapter sequences were removed through quality filtering and trimming to obtain high-quality clean reads. After filtering, 56.90 million clean reads were retained for NHN, 80.98 million for Pink+4 (#20A09), 50.83 million for the low-CRE bulk, and 48.40 million for the high-CRE bulk (**Supplementary Table 2**).

Clean reads from each sample were aligned to the Nipponbare reference genome. The alignment generated a total of 8.53 Gb of clean sequence data corresponding to approximately 19.81× genome coverage for NHN, 12.05 Gb (25.65× coverage) for Pink+4 (#20A09), 7.54 Gb (15.18× coverage) for the low-CRE bulk, and 7.18 Gb (15.84× coverage) for the high-CRE bulk (**Supplementary Table 2**).

High-quality reads obtained from NHN were used to construct the reference sequence for subsequent QTL-seq analysis. Clean reads from the high-CRE and low-CRE bulks were aligned to the NHN reference sequence, resulting in the identification of 1,234,622 sequence variants, including single-nucleotide polymorphisms (SNPs) and insertion/deletion polymorphisms (InDels), supported by at least three sequencing reads in both bulks (**Supplementary Table 3**).

To improve the reliability of variant detection, only SNPs supported by a minimum read depth of 18 reads were retained for further analysis. Using this criterion, 18,774 high-confidence SNPs and InDels were selected for SNP index calculation in each bulk (**Supplementary Table 3**). SNPs exhibiting SNP index values lower than 0.3 in both bulks were filtered out to minimize false-positive variants potentially arising from sequencing or read alignment errors.

### 3.4 Candidate regions for cooked rice elongation

**Figure 2:**
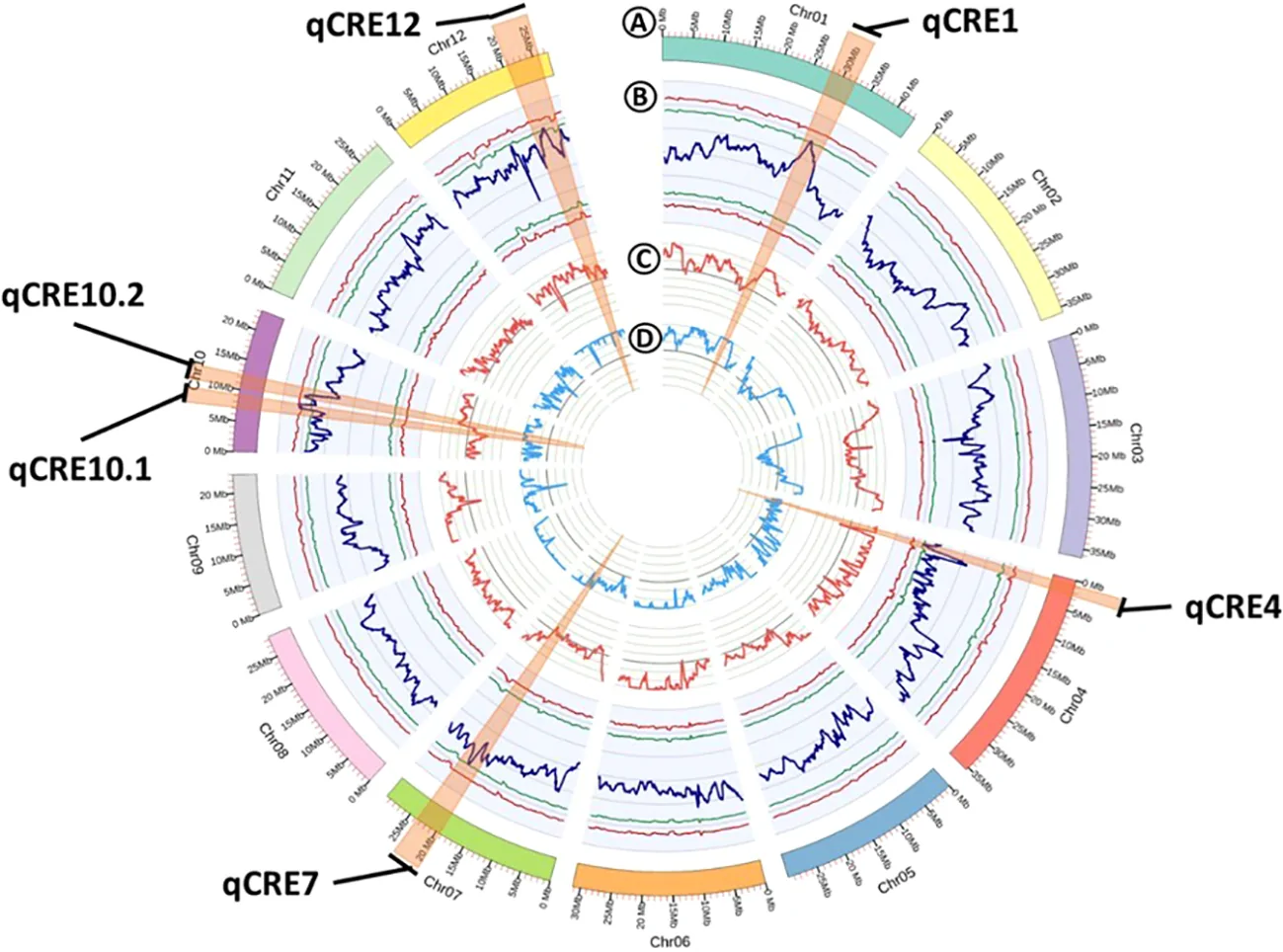
Δ(SNP index) compared between the SNP index plot of two bulks (high-CRE bulk and low-CRE bulk). **(A)** Pseudomolecules of the Nipponbare reference genome (IRGSP 1.0). **(B)** Comparison of Δ(SNP index) between the two bulks. Green and red borders indicate regions with highly significant p-values at the 95% and 99% confidence levels, respectively. **(C)** SNP index of low-CRE bulk. **(D)** SNP index of high-CRE bulk. The sliding window plots of average SNP index values with a 2-Mb window size and 10-kb steps. Candidate genomic regions containing QTLs for CRE are shown in triangle areas.

**Figure 3:**
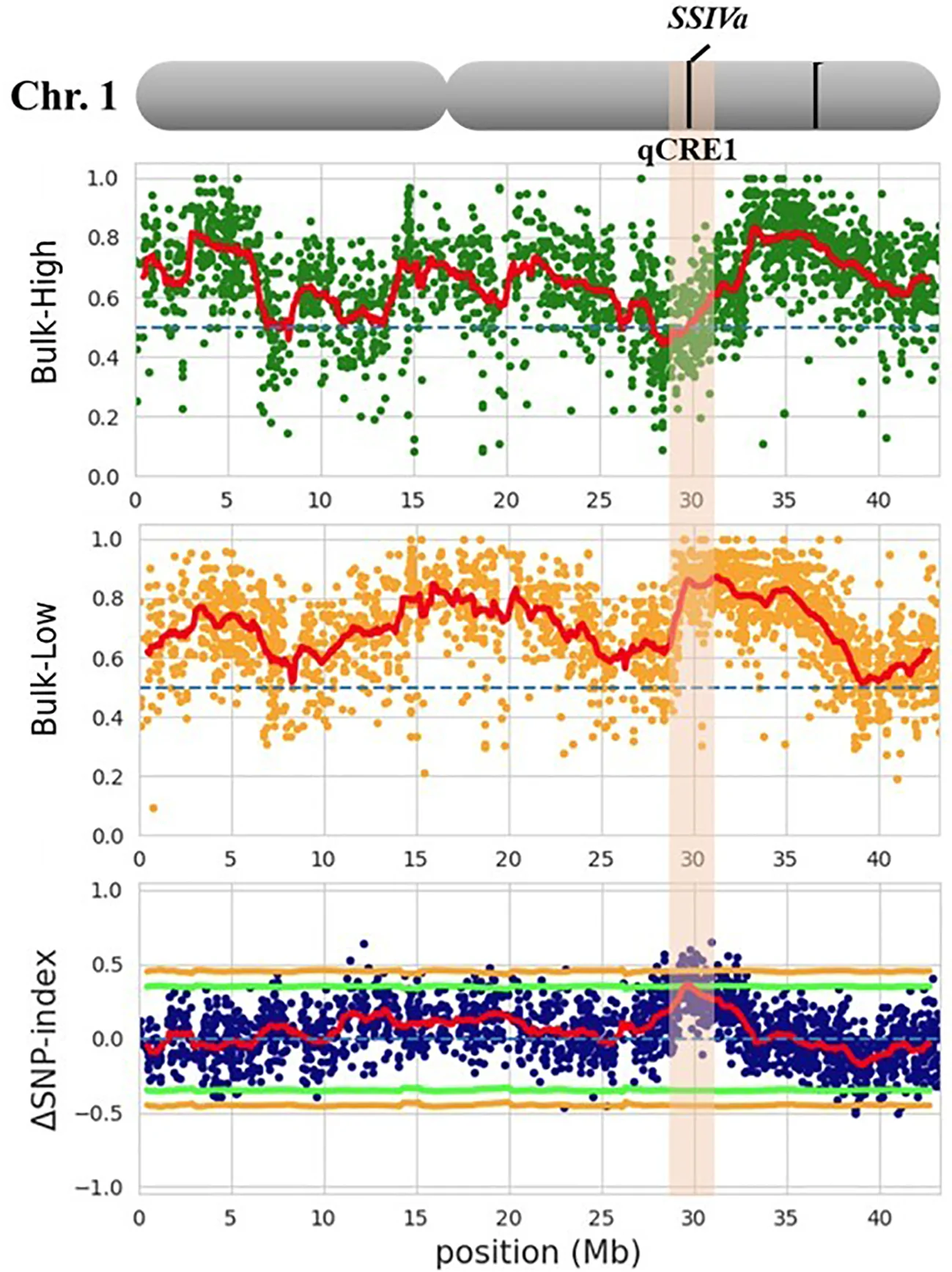
A highly significant Δ(SNP-index) region was identified on chromosome 1 between 29.3 and 30.2 Mb.

For the ΔSNP index between the opposite CRE bulks, we identified six critical genomic regions linked with CRE on chromosomes 1 (*qCRE1*), 4 (*qCRE4*), 7 (*qCRE7*), 10 (*qCRE10.1* and *qCRE10.2*), and 12 (*qCRE12*) (**Figure 2**; **Table 1**). In addition, the *qCRE1*, *qCRE4*, *qCRE7*, *qCRE10.1*, *qCRE10.2* and *qCRE12* were specified within the 29.3-30.2 Mb, 1.76-1.85 Mb, 21.4-21.8 Mb, 7.3-9.8 Mb, 10.2-11.3 Mb and 21.7-25.8 Mb genomic regions, respectively (**Table 1**; **Figure 3**; **Supplementary Figure 2**-**5**). Initially, we listed all 104 candidate genes localized within the peaks of the ΔSNP indexes using the Rice Annotation Project Database (RAP-DB, http://rapdb.dna.affrc.go.jp/) (**Supplementary Tables 4**, **5**). Only 26 candidate genes, for which ΔSNP indexes > 0.5, were used to develop KASP markers, including nine markers for *qCRE1*, one for *qCRE4*, two for *qCRE7*, 12 for *qCRE10.1* and *qCRE10.2*, and two for *qCRE12* (**Table 2**; **Supplementary Tables 4**, **5**). The 105 BC_1_F_2_ individuals were genotyped and phenotyped for CRE and associated traits using BC_1_F_2_-derived F_3_ seeds (**Table 2**, **Supplementary Tables 6**, **7**). Single-marker analysis revealed only *qCRE1* was statistically significant based on the R-square value (≥15%PVE), F-test, and P-value <0.01 (**Table 2**; **Supplementary Table 5**).

**Table 1:** Summary of candidate genomic regions associated with cooked rice CRE identified by QTL-seq analysis.

QTL	Chr.	Region (Mb)	Interval (Mb)	Peak (Mb)	Confidence interval (95%)
*qCRE1*	1	29.3-30.2	0.9	30.1	0.3
*qCRE4*	4	1.76-1.85	0.9	1.84	0.3
*qCRE7*	7	21.4-21.8	0.4	21.6	0.3
*qCRE10.1*	10	7.3-9.8	2.5	8.9	0.3
*qCRE10.2*	10	10.2-11.3	1.1	11.2	0.3
*qCRE12*	12	21.7-25.8	4.1	22.9	0.3

**Table 2:** Single-marker analysis of 26 SNP markers associated with CRE in the BC_1_F_2_ population based on SNP Indexes ≥ 0.5 (P-value > 99%).

Marker_name	Chr.	R_square	%PVE	F-test	P-value	Gene ID	Description
R0129312181	1	0.166950	16.70	22.2453	7.03E-06	Os01g0706400	DUF292 domain containing protein.
R0129322283	1	0.159501	15.95	21.0645	1.18E-05	Os01g0706700	DUF803 domain containing protein.
R0129324976	1	0.167848	16.78	22.3891	6.60E-06	Os01g0706700	DUF803 domain containing protein.
R0129733026	1	0.171654	17.17	23.0020	5.07E-06	Os01g0715000	Conserved hypothetical protein.
R0129771713	1	0.152483	15.25	19.9709	1.90E-05	Os01g0715600	Auxin efflux carrier component.
R0129975201	1	0.176576	17.66	23.8030	3.59E-06	Os01g0719100	CHY zinc finger domain-containing protein 1.
R0130038156	1	0.289328	28.93	45.1903	8.06E-10	Os01g0720600	Starch synthase IVa.
R0130057841	1	0.235752	23.58	34.2408	5.01E-08	Os01g0720801	Hypothetical gene.
R0130196202	1	0.212845	21.28	30.0142	2.71E-07	Os01g0723800	P-glycoprotein homologue.
R0401849181	4	0.000710	0.07	0.0597	0.8075	Os04g0129350	Hypothetical gene.
R0721413833	7	0.019988	2.00	2.2640	0.1353	Os07g0541800	KI domain interacting kinase 1.
R0721599574	7	0.000035	0.00	0.0039	0.9504	Os07g0545500	VMP4 protein.
R1007303735	10	0.008366	0.84	0.9365	0.3353	Os10g0202501	Hypothetical gene.
R1008803290	10	0.004818	0.48	0.5374	0.4651	Os10g0321700	Ribonucleoprotein like protein.
R1008869299	10	0.009092	0.91	1.0185	0.3151	Os10g0322600	Hypothetical gene.
R1008935229	10	0.012758	1.28	1.4344	0.2336	Os10g0323900	Profilin A.
R1008984630	10	0.020151	2.02	2.2827	0.1337	Os10g0324600	C_2_H_2_-like domain containing protein.
R1009195457	10	0.015787	1.58	1.7805	0.1848	Os10g0328100	Hypothetical protein.
R1009902249	10	0.037496	3.75	4.3242	0.0399	Os10g0339290	Alpha-1,2-fucosidase.
R1010217652	10	0.011145	1.11	1.2510	0.2658	Os10g0343900	Thioredoxin fold domain containing protein.
R1011089916	10	0.016217	1.62	1.8298	0.1789	Os10g0360800	LRR-receptor-like kinase family protein.
R1011255488	10	0.023824	2.38	2.7090	0.1026	Os10g0363100	glycosyltransferase, family GT8
R1011259388	10	0.018228	1.82	2.0609	0.1539	Os10g0363100	glycosyltransferase, family GT8
R1011274471	10	0.027843	2.78	3.1791	0.0773	Os10g0363300	Acetyl-CoA carboxylase.
R1222020155	12	0.010425	1.04	1.1694	0.2819	Os12g0545600	Hypothetical protein.
R1223010774	12	0.006902	0.69	0.7714	0.3817	Os12g0561750	Ferroportin protein family.

### 3.5 Identification of informative SNPs affecting CRE

**Figure 4:**
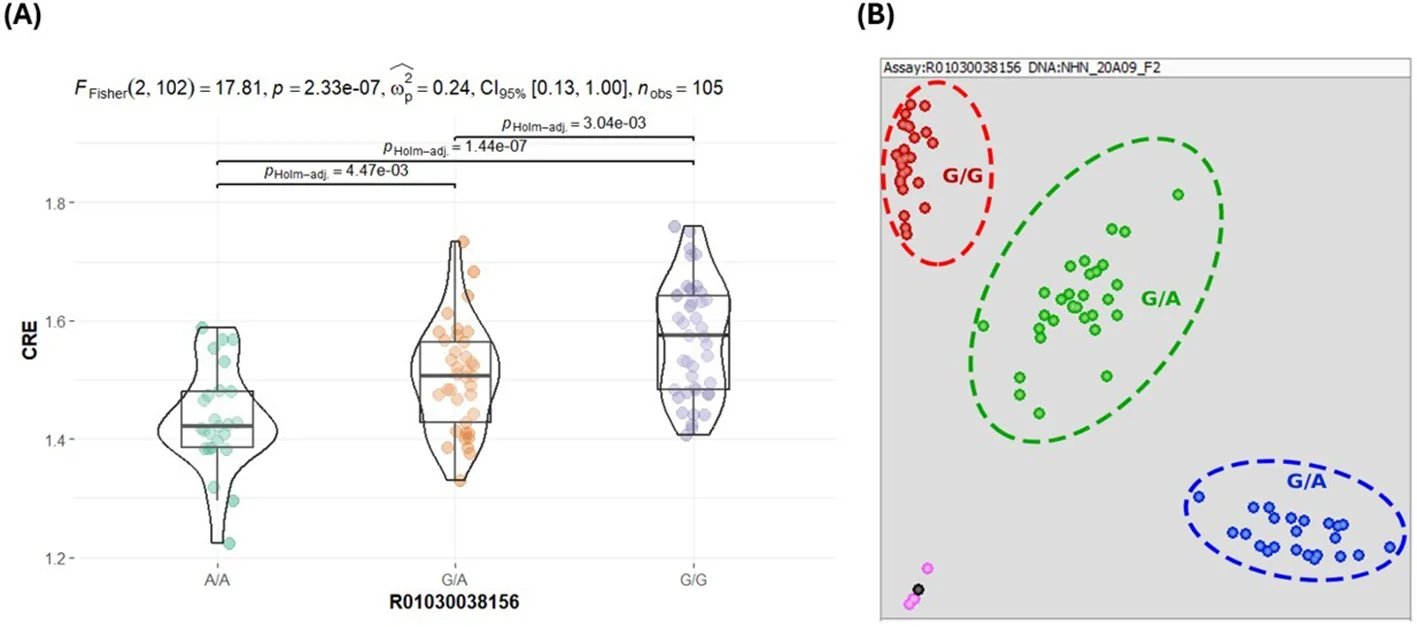
**(A)** The impact of *SSIVa*_*1_30038156* (R0130038156) on the CRE ratio from the 105 BC_1_F_2_ mapping population. P-value ≤0.01 indicates a significant level of allele-specific marker. **(B)** Genotype-based calling of *SSIVa*_*1_30038156* (R0130038156) based on allele-specific fluorescence clustering.

**Figure 5:**
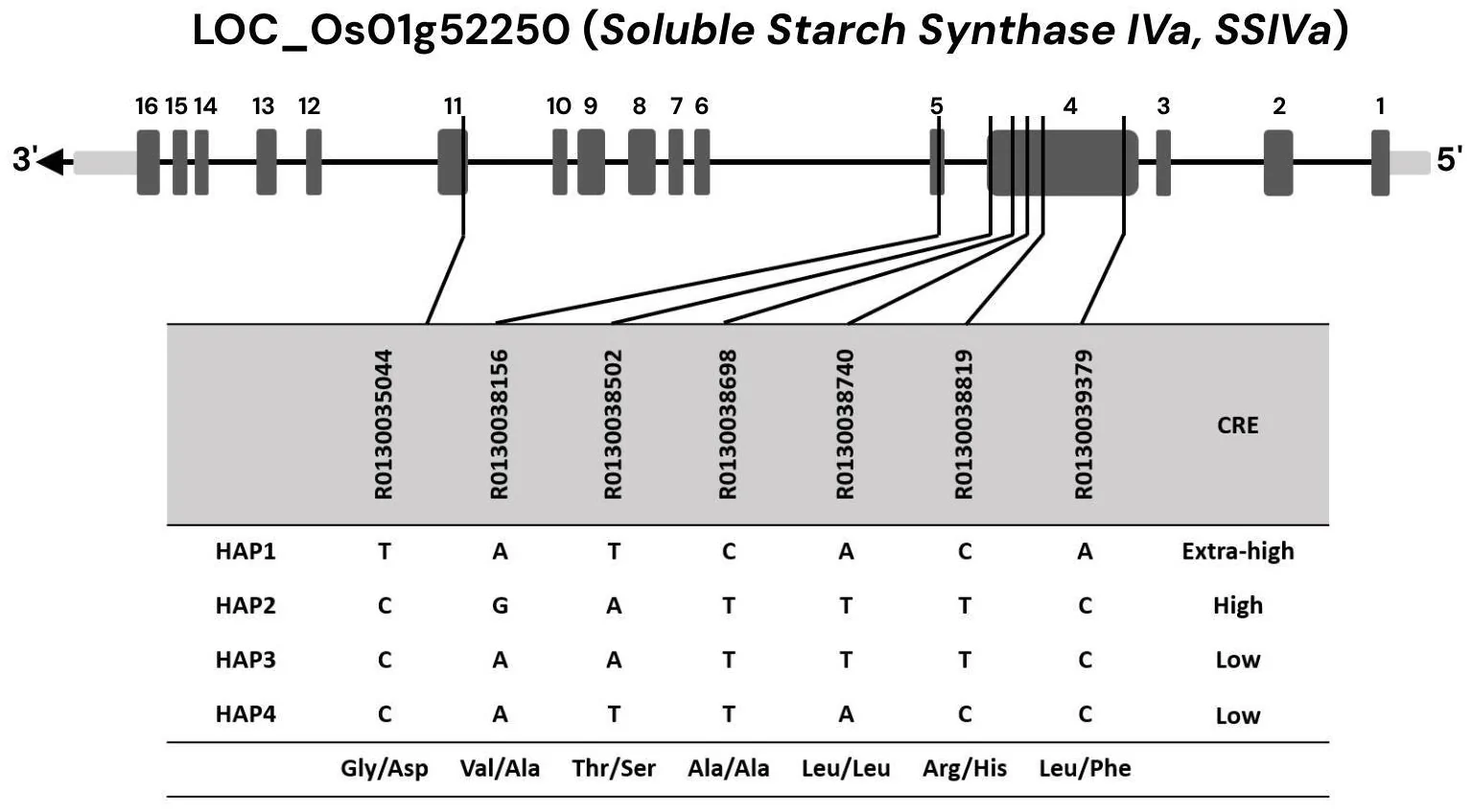
Positions (3′-5′) of informative SNPs with genomic coordinates on three exons of *SSIVa* and four haplotypes associated with CRE based on 31 diverged waxy and non-waxy rice varieties.

Within *qCRE1*, nine significantly different SNPs were identified, but only R0130038156 (S156), localized within Exon 5 of Os01g0720600 (LOC_Os01g52250), gained almost 30% of the PVE (**Table 2**; **Figure 4**; **Supplementary Table 5**). The candidate gene is *Soluble Starch Synthase IVa* (*SSIVa*), which is the only starch biosynthetic gene in the genomic region. Within the coding sequence, we identified six additional SNPs from NSTDA Crop Database Genome Browser (https://sinin.kps.ku.ac.th/ricedbtools/jbrowse/) (**Supplementary Table 6**): *SSIVa_1_30035044* (S044, Exon 11). *SSIVa_1_30038502* (S502, Exon 4), *SSIVa_1_30038698* (S698, Exon 4), *SSIVa_1_30038740* (S740, Exon 4), *SSIVa_1_30038819* (S819, Exon 4), and *SSIVa_1_30039379* (S379, Exon 4). Although S044 and S698 were monomorphic between the parents, they were polymorphic among the germplasm. The mutation at S156 is a non-synonymous change from G to A, causing the amino acid change from Val to Ala, while S044 (Gly/Asp), S502 (Thr/Ser), S819 (Arg/His), and S379 (Leu/Phe) were also non-synonymous. Only S698 and S740 were synonymous (**Table 3**; **Figure 5**). In total, seven informative SNPs were selected for further analysis.

**Table 3:** Genotypes of 31 diverged rice varieties based on seven polymorphic SNPs for *SSIVa* on chromosome 1, one SNP and 23 bp indel for *GBSSI*, and two SNPs for *SSIIa* on chromosome 6. CRE, Grain type, and Haplotype are shown for each variety.

Name	R0130035044	R0130038156	R0130038502	R0130038698	R0130038740	R0130038819	R0130039379	R0601765761	R0601767006	R0606752887	R0606752888	CRE	Grain type	Haplotype
Paw San Hmwe	T	A	T	C	A	C	A	G	–	G	C	2.04 ± 0.11	Nw,S,W	HAP1
PB1885_153	T	A	T	C	A	C	A	G	–	G	C	1.91 ± 0.10	Nw,L,W	
PB1885_154	T	A	T	C	A	C	A	G	–	G	C	1.91 ± 0.06	Nw,L,W	
Pusa Basmati_1121	T	A	T	C	A	C	A	G	–	T	T	2.04 ± 0.10	Nw,L,W	
PB1121_149	T	A	T	C	A	C	A	G	–	T	T	1.98 ± 0.12	Nw,L,W	
PB1121_150	T	A	T	C	A	C	A	G	–	T	T	2.06 ± 0.12	Nw,L,W	
PB1401_146	T	A	T	C	A	C	A	G	–	T	T	2.16 ± 0.10	Nw,L,W	
PB1401_145	T	A	T	C	A	C	A	G	–	T	T	1.96 ± 0.08	Nw,L,W	
Pusa Basmati_1718	T	A	T	C	A	C	A	G	–	T	T	2.06 ± 0.15	Nw,L,W	
PB1718_155	T	A	T	C	A	C	A	G	–	T	T	2.04 ± 0.12	Nw,L,W	
PB1718_156	T	A	T	C	A	C	A	G	–	T	T	1.94 ± 0.08	Nw,L,W	
PB1509_147	C	G	A	T	T	T	C	G	–	T	T	1.84 ± 0.12	Nw,L,W	HAP2
PB1509_148	C	G	A	T	T	T	C	G	–	T	T	1.79 ± 0.10	Nw,L,W	
PB1692_152	C	G	A	T	T	T	C	G	–	T	T	1.86 ± 0.07	Nw,L,W	
NHN	C	G	A	T	T	T	C	T	23 dup	G	C	1.72 ± 0.10	Wx,L,W	
PB1692_151	C	G	A	T	T	T	C	T	–	T	T	1.83 ± 0.08	Nw,L,W	
Niaw Luem Pua	C	G	A	T	T	T	C	T	23 dup	T	T	1.70 ± 0.11	Wx,L,P	
Niaw Dam Chaw Mai Pai_L	C	G	A	T	T	T	C	T	23 dup	T	T	1.76 ± 0.16	Wx,L,P	
Niaw Dam Cham Mai Pai_W	C	A	A	T	T	T	C	T	23 dup	T	T	1.57±0.04	Wx,S,P	
Jao Khao Chiang Mai	C	A	A	T	T	T	C	G	–	G	C	1.55 ± 0.12	Nw,L,W	HAP3
I Thi	C	A	A	T	T	T	C	T	23 dup	T	T	1.54 ± 0.09	Wx,S,W	
Khao Niaw Dam	C	A	A	T	T	T	C	T	23 dup	T	T	1.33 ± 0.06	Wx,L,P	
Niaw Dam Maw 37	C	A	A	T	T	T	C	T	23 dup	T	T	1.37 ± 0.04	Wx,L,P	
Hom Bao Rai	C	A	A	T	T	T	C	G	–	G	C	1.54 ± 0.09	Nw,L,W	
Num Roo	C	A	A	T	T	T	C	G	–	G	C	1.46 ± 0.08	Nw,L,W	
Khao Kai Noi Leuang	C	A	A	T	T	T	C	T	23 dup	G	C	1.52 ± 0.09	Nw,S.W	
Dok Kha 50	C	A	T	T	A	C	C	G	–	G	C	1.58 ± 0.07	Nw,L,W	HAP4
DH103	C	A	T	T	A	C	C	G	–	G	C	1.59 ± 0.08	Nw,L,W	
Med Fay 62	C	A	T	T	A	C	C	G	–	G	C	1.46 ± 0.06	Nw,L,W	
Pink+4 (#20A09)	C	A	T	T	A	C	C	G	–	T	T	1.37 ± 0.08	Nw,L,W	
PTT1	C	A	T	T	A	C	C	T	–	T	T	1.42 ± 0.04	Nw,L,W	

Grain types: Nw, non-waxy; Wx, waxy; S, short grain; L, long grain; W, non-pigmented; P, pigmented.

In addition, previously characterized functional SNP markers previously reported to associated with CRE were included for comparison: two SNPs of *Granule Bound Starch Synthase I* (*GBSSI*; Os06g0133000), which controls amylose content, and *Starch Synthase IIa* (*SSIIa*; two SNPs of Os06g0229900) (Arikit et al., 2019) (**Table 3**). The 11 SNP markers included seven for *SSIVa* on chromosome 1 and two each for *GBSSI* and *SSIIa* on chromosome 6 were used to screen all 31 rice varieties with known CRE and associated traits (**Table 3**).

### 3.6 Informative haplotypes associated with CRE

To identify haplotypes associated with *SSIVa* and CRE, we selected 31 rice varieties across a broader spectrum of waxy and non-waxy with known and unknown CRE and genotyped them using the seven polymorphic SNPs within the *SSIVa*, including five on exon 4 and one each on exon 5 and exon 11 (**Figure 5**; **Supplementary Table 8**; **Supplementary Figure 6**). Two informative SNPs each for *GBSSI* and *SSIIa* were also included in the genotyping. In total, we used 11 SNPs to search for haplotypes associated with CRE (**Figure 5**). We identified four haplotypes formulated only from the seven SNPs in *SSIVa* were associated with CRE, whereas four SNPs from *GBSSI* and *SSIIa* were not considered (**Table 3**). The four haplotypes are Hap 1: T-A-T-C-A-C-A, Hap 2: C-G-A-T-T-T-C, Hap 3: C-A-A-T-T-T-C, and Hap 4: C-A-T-T-A-C-C. The HAP1 included seven rice varieties, which show extra-high-CRE, ranging from 1.81 to 2.16, and were mostly Basmati and Paw San Hmwe. The HAP2 included seven rice varieties, comprising both waxy and non-waxy types, which show high-CRE values, ranging from 1.7 to 1.86, and were three waxy rice varieties, including NHN, Niaw Dam Chaw Mai Pai (Pigmented), and Niaw Luem Pua (Pigmented), and four Basmati accessions, including PB1590-147, PB1590-148, PB1692-151, and PB1692-152 (**Table 3**). The low-CRE rice varieties, ranging from 1.3 to 1.5, were included in two haplotypes, HAP3 and HAP4. The HAP3 rice varieties comprise waxy, non-waxy, and pigmented varieties, whereas the HAP4 varieties are all non-waxy. These haplotypic markers are useful for future marker-assisted selection to improve elite rice varieties with CRE (**Table 3**).

## 4 Discussion

### 4.1 Surprising roles of *SSIVa*

This study was initially designed to identify genes underlying cooked-rice elongation (CRE) in NHN, an extra-long-grain, high-CRE waxy rice genotype characterized by a high gelatinization temperature (GT). Such a combination of waxy endosperm, high-GT, and pronounced cooked-grain elongation is relatively uncommon among traditional rice varieties. Previous QTL and association studies have identified major loci associated with CRE and related grain-quality traits near starch-biosynthesis genes, including *GBSSI*, *SSIIa*, *SBEIIb*, and *GS3*, as well as several novel genomic regions across different chromosomes. However, *SSIVa* has not previously been identified as a major candidate gene associated with CRE.

One possible explanation for the absence of *SSIVa* in previous studies is the difference in genetic backgrounds and phenotypic characteristics among the rice materials investigated. Many previous studies of high-CRE rice focused on high-amylose genotypes associated with *GBSSI* and high-GT genotypes associated with *SSIIa*, including Basmati, Zhenshan 97, and Paw San Hmwe (Qiu et al., 2021; Arikit et al., 2019; Zhang et al., 2004; Ge et al., 2005; Malik et al., 2022; Yang et al., 2013; Ahn et al., 1993; Govindaraj et al., 2009; Tian et al., 2005; Ramkumar et al., 2010; Wang et al., 2007; Thi et al., 2020; Amarawathi et al., 2008; Fujita et al., 2022; Johar and Salgotra, 2025). In contrast, our analysis did not provide evidence for a major contribution of *SSIIa* to CRE in the present population. Instead, our results identified *SSIVa* as a strong candidate associated with CRE variation in both waxy and non-waxy rice.

Four *SSIVa* haplotypes differentiated the rice germplasm into three CRE groups: HAP1 (extra-high-CRE), HAP2 (high-CRE), and HAP3/HAP4 (low-CRE). NHN was classified as HAP2 and exhibited a CRE value of 1.72. Nevertheless, its relatively long grain length before cooking resulted in an extra-long cooked grain, with a cooked grain length exceeding 1.8. These findings suggest that both the *SSIVa* haplotype and the initial grain dimensions may contribute to the final extent of cooked-grain elongation.

Interestingly, a similar *qCRE1* region may also have been detected in the QTL-seq analysis of the Basmati × PTT1 population reported by Arikit et al. (2019). Although the strongest ΔSNP-index signal in that study was located on chromosome 6 near *SSIIa* (*qGE6.1*), a weaker and statistically non-significant signal was also observed in a region that co-localized with *qCRE1* identified in our NHN × Pink+4 (#20A09) population. This observation raises the possibility that chromosome 1 may contribute to cooked-grain elongation across genetically diverse rice backgrounds, although the magnitude and significance of its effect may depend on the genetic background and allelic composition of the population.

### 4.2 *SSIVa*: multi-task enzyme

CRE is associated with high longitudinal expansion and water absorption rate during cooking (Juliano and Perez, 1984). *SSIVa* is a cereal starch synthase isoform involved in starch biosynthesis and granule architecture (Hirose and Terao, 2004). Relative to *SSI*, *SSIIa*, and *SSIIIa*, *SSIVa* influences granule initiation and number: loss of function lines often produce fewer, larger granules with no major changes in total polysaccharide composition (Roldán et al., 2007; Irshad et al., 2021). Allelic variation in *SSIVa* correlates with differences in the physicochemical properties of starch, including gelatinization and thermal behavior, which can affect eating quality (Xu et al., 2020). *SSIVa* participates in protein–protein interactions that help assemble multi-subunit biosynthetic complexes, shaping amylopectin structure (Ida et al., 2022; Zhang et al., 2021). Evidence from rice indicates that factors other than *SSIVa*/*SSIVb* also contribute to endosperm granule initiation (Toyosawa et al., 2016). Further genetic and functional studies in rice are needed to define a precise role of *SSIVa* in granule biogenesis and grain quality traits such as CRE.

Recently, a GWAS across 345 diverse rice accessions identified multiple starch related loci associated with grain length/breadth expansion and revealed substantial genotype × environment effects (Zheng et al., 2023). The GWAS prioritized distinct starch/endosperm genes (*SSIIIb*, LOC_Os04g53310; *MT2b*, LOC_Os05g02070; *GBSSI*, LOC_Os06g04200; *SSIIa*, LOC_Os06g12450; and *SSIIIa*, LOC_Os08g09230) based on haplotype and expression evidence, with top QTNs concentrated on chromosomes 4, 5, 6, and 8, respectively. In another GWAS for root architecture, 12 non-synonymous SNPs were identified within the coding region of *SSIVa*. Sequence variations in this gene have been linked to root architecture traits and starch biosynthesis/quality (Xiang et al., 2022; Zhao et al., 2021). Five distinct haplotypes (HapA-HapE) directly influence Main Root Length (MRL) and Average Root Length (ARL) (Xiang et al., 2022). The fact that only five out of seven SNPs in our study overlap with the root architecture study, *SSIVa* may play a vital role in cellular elongation and possibly storage of starch and polysaccharides within the root. In addition to major loci such as *Wx*, allelic variations in *SSIVa* contribute to quantitative trait loci (QTLs) for RVA (Rapid Visco Analyser) profiles and paste viscosity (Zhao et al., 2021).

In contrast, our QTL-seq analysis uncovered several high-effect non-synonymous variants in *SSIVa* (LOC_Os01g52250) within *qCRE1*, which were strongly associated with cooked-rice elongation (CRE) in the waxy rice genotype NHN. Future studies must focus on how multiple non-synonymous mutations in *SSIVa* affect the roles of *SSIVa* in shaping cellular elongation, starch granule architecture, and starch storage across different organs. Previous transcriptomic studies have demonstrated that *SSIVa* is expressed during rice grain development and that its transcript abundance varies depending on genotype and developmental stage. Therefore, although the polymorphisms identified in the present study are located within the coding region, an effect of the *SSIVa* haplotypes on transcript abundance cannot be completely excluded. Differences in *SSIVa* expression between contrasting CRE haplotypes may contribute to the observed phenotypic variation. However, whether the identified polymorphisms directly affect transcript abundance remains to be determined. Further comparative expression analysis, particularly between high- and low-CRE haplotypes during grain development, would be required to clarify this possibility.

## 5 Conclusion

We successfully identified *SSIVa* as the key genetic determination of cooked rice elongation (CRE) in rice. Eleven polymorphic SNPs in the coding sequence of *SSIVa* consist of seven non-synonymous, four synonymous, and one in-frame deletion mutation among 31 diverged waxy and non-waxy rice varieties. Four haplotypes, HAP 1, 2, 3, and 4, identified from the seven informative SNPs classified the germplasm into Extra-, High-, and Low-CRE groups. HAP1 contains world-renowned CRE rice varieties, including 11 Basmati and the small-grain Paw San from Myanmar, with CRE >1.9. HAP2 comprises three waxy and four Basmati rice varieties, with CRE between 1.7 and 1.86. HAP3–4 included five waxy and eight non-waxy rice varieties, with CRE < 1.59. Haplotypic markers derived from these seven informative SNPs can be effectively used to screen desirable germplasm and to support marker-assisted selection aimed at improving CRE rice with high precision.

## Data Availability

The original contributions presented in the study are publicly available. This data can be found here: NCBI, PRJNA1522849.
