# Supplementary material for: Soluble starch synthase IVa determines cooked rice elongation in waxy and non-waxy rices (Oryza sativa L.)
Source: Front Plant Sci. 2026 Sep 11;17:1922515. doi: 10.3389/fpls.2026.1922515 (PMC13612430; doi:10.3389/fpls.2026.1922515)
Supplement: Supplementary file 1 [file DataSheet1.docx]

Supplementary Material

# 1 Supplementary Data

## 1.1 Supplementary Tables

**Supplementary Table 1.** CRE ratios of the parental lines, NHN and Pink+4 (#20A09), and the BC_1_F_2_-derived F_3_ seeds population used for phenotypic evaluation and QTL-seq analysis.

| **No.** | **name** | **L0 (mm)** | | | | | **AVG** | **L1 (mm)** | | | | | **AVG** | **Ratio (L1-L0)** |
| --- | --- | --- | --- | --- | --- | --- | --- | --- | --- | --- | --- | --- | --- | --- |
|  |  | **S1** | **S2** | **S3** | **S4** | **S5** |  | **S1** | **S2** | **S3** | **S4** | **S5** |  |  |
| - | Pink+4 (#20A09) | 7.40 | 7.40 | 7.41 | 7.46 | 7.44 | 7.42 | 10.11 | 10.14 | 10.15 | 10.15 | 10.15 | 10.14 | 1.37 |
| - | NHN | 8.43 | 8.47 | 8.48 | 8.40 | 8.41 | 8.44 | 14.60 | 14.43 | 14.41 | 14.43 | 14.50 | 14.47 | 1.72 |
| #1 | 01_A06_50_03 | 7.45 | 7.68 | 6.70 | 7.58 | 7.38 | 7.36 | 10.28 | 10.53 | 10.58 | 10.38 | 10.51 | 10.46 | 1.42 |
| #2 | 01_A06_50_05 | 7.60 | 7.58 | 7.35 | 7.95 | 7.90 | 7.67 | 12.22 | 13.03 | 13.00 | 12.46 | 12.39 | 12.62 | 1.64 |
| #3 | 01_A06_50_06 | 7.69 | 8.22 | 8.32 | 7.99 | 8.33 | 8.11 | 12.70 | 13.84 | 14.66 | 12.07 | 13.05 | 13.26 | 1.64 |
| #4 | 01_A07_51_01 | 8.07 | 8.07 | 8.17 | 8.50 | 8.01 | 8.16 | 11.36 | 11.55 | 10.86 | 11.89 | 11.91 | 11.51 | 1.41 |
| #5 | 01_A07_51_02 | 8.83 | 8.67 | 8.44 | 8.98 | 8.85 | 8.76 | 14.20 | 13.89 | 13.78 | 13.90 | 13.46 | 13.85 | 1.58 |
| #6 | 01_A07_51_04 | 7.01 | 7.17 | 7.19 | 6.70 | 7.10 | 7.03 | 11.86 | 11.51 | 11.20 | 11.66 | 11.18 | 11.48 | 1.63 |
| #7 | 01_A07_51_05 | 7.24 | 7.18 | 7.57 | 7.25 | 7.35 | 7.32 | 11.80 | 11.21 | 11.64 | 11.60 | 11.81 | 11.61 | 1.59 |
| #8 | 01_A07_51_09 | 7.38 | 7.23 | 7.49 | 6.77 | 7.24 | 7.22 | 11.34 | 11.48 | 11.49 | 11.37 | 10.81 | 11.30 | 1.56 |
| #9 | 01_B04_34_04 | 6.08 | 5.88 | 5.85 | 5.63 | 6.07 | 5.90 | 10.26 | 9.92 | 10.93 | 10.73 | 9.85 | 10.34 | 1.75 |
| #10 | 01_B04_34_13 | 6.90 | 6.90 | 7.59 | 6.86 | 7.16 | 7.08 | 10.61 | 10.46 | 10.54 | 9.79 | 10.55 | 10.39 | 1.47 |
| #11 | 01_D11_34_01 | 8.22 | 8.47 | 8.87 | 8.43 | 7.82 | 8.36 | 11.48 | 12.08 | 11.81 | 11.33 | 11.25 | 11.59 | 1.39 |
| #12 | 01_D11_34_06 | 6.13 | 6.84 | 6.72 | 7.15 | 7.08 | 6.78 | 9.58 | 10.26 | 10.61 | 11.13 | 11.79 | 10.67 | 1.57 |
| #13 | 01_D11_34_10 | 8.58 | 8.50 | 8.57 | 8.26 | 8.68 | 8.52 | 11.17 | 12.12 | 12.12 | 11.98 | 11.62 | 11.80 | 1.39 |
| #14 | 01_E12_18_01 | 7.87 | 7.91 | 7.74 | 7.36 | 7.68 | 7.71 | 12.25 | 11.96 | 11.87 | 12.15 | 12.51 | 12.15 | 1.58 |
| #15 | 01_E12_18_07 | 7.50 | 7.58 | 7.45 | 7.40 | 7.90 | 7.56 | 12.12 | 13.03 | 12.90 | 12.46 | 12.04 | 12.51 | 1.65 |
| #16 | 01_E12_18_08 | 6.86 | 6.88 | 6.64 | 7.06 | 7.31 | 6.95 | 10.27 | 10.99 | 10.28 | 10.00 | 9.99 | 10.30 | 1.48 |
| #17 | 01_E12_18_10 | 7.71 | 7.60 | 8.01 | 8.26 | 8.34 | 7.98 | 11.36 | 11.92 | 12.16 | 12.81 | 12.55 | 12.16 | 1.52 |
| #18 | 01_F05_56_01 | 7.65 | 7.82 | 7.42 | 8.03 | 7.60 | 7.70 | 10.39 | 11.37 | 10.20 | 10.56 | 10.74 | 10.65 | 1.38 |
| #19 | 01_F05_56_04 | 7.98 | 7.13 | 7.63 | 7.40 | 7.76 | 7.58 | 12.29 | 13.30 | 12.89 | 13.78 | 13.49 | 13.15 | 1.73 |
| #20 | 01_F05_56_06 | 6.83 | 7.25 | 7.08 | 7.96 | 7.54 | 7.33 | 9.08 | 9.58 | 9.49 | 9.53 | 9.83 | 9.50 | 1.30 |
| #21 | 01_F05_56_07 | 7.86 | 7.90 | 7.52 | 7.37 | 7.13 | 7.55 | 12.10 | 12.01 | 11.61 | 12.28 | 12.31 | 12.06 | 1.60 |
| #22 | 01_F05_56_08 | 7.44 | 7.18 | 7.77 | 7.25 | 7.35 | 7.40 | 11.80 | 11.21 | 11.64 | 11.60 | 11.81 | 11.61 | 1.57 |
| #23 | 01_G10_53_04 | 8.00 | 8.75 | 8.60 | 8.32 | 8.62 | 8.46 | 13.52 | 13.61 | 13.42 | 13.32 | 13.34 | 13.44 | 1.59 |
| #24 | 01_G10_53_05 | 7.15 | 5.98 | 6.15 | 6.53 | 7.02 | 6.57 | 10.17 | 9.99 | 9.24 | 10.12 | 10.46 | 10.00 | 1.52 |
| #25 | 01_G10_53_06 | 7.40 | 6.91 | 6.85 | 7.07 | 7.35 | 7.11 | 11.48 | 11.61 | 11.10 | 10.94 | 11.13 | 11.25 | 1.58 |
| #26 | 01_G10_53_07 | 7.49 | 7.25 | 7.44 | 7.35 | 7.24 | 7.35 | 12.36 | 12.31 | 11.78 | 12.28 | 12.24 | 12.19 | 1.66 |
| #27 | 01_G10_53_08 | 8.26 | 8.68 | 8.32 | 8.89 | 8.27 | 8.49 | 12.44 | 12.64 | 12.76 | 12.54 | 12.19 | 12.51 | 1.47 |
| #28 | 01_G10_53_09 | 7.75 | 7.92 | 7.32 | 8.03 | 7.50 | 7.70 | 10.29 | 11.27 | 10.10 | 10.66 | 10.64 | 10.59 | 1.38 |
| #29 | 02_A02_49_01 | 6.66 | 7.17 | 6.68 | 7.25 | 6.88 | 6.93 | 10.01 | 10.90 | 9.92 | 10.15 | 10.26 | 10.25 | 1.48 |
| #30 | 02_A02_49_01 | 7.71 | 7.63 | 7.45 | 7.39 | 7.43 | 7.52 | 12.68 | 11.90 | 11.90 | 10.74 | 12.54 | 11.95 | 1.59 |
| #31 | 02_A02_49_03 | 6.60 | 6.78 | 7.51 | 7.95 | 7.08 | 7.18 | 10.41 | 10.02 | 11.31 | 11.43 | 10.55 | 10.74 | 1.50 |
| #32 | 02_A02_49_08 | 8.58 | 8.50 | 8.66 | 8.26 | 8.68 | 8.54 | 12.17 | 12.12 | 12.12 | 11.98 | 11.62 | 12.00 | 1.41 |
| #33 | 02_D03_45_04 | 7.45 | 7.16 | 7.50 | 7.48 | 8.09 | 7.53 | 11.49 | 10.86 | 10.24 | 10.77 | 10.12 | 10.69 | 1.42 |
| #34 | 02_D03_45_05 | 6.56 | 7.08 | 6.83 | 6.96 | 6.53 | 6.79 | 10.83 | 10.67 | 10.44 | 10.08 | 10.77 | 10.56 | 1.55 |
| #35 | 02_D07_44_01 | 8.94 | 8.43 | 8.31 | 8.57 | 8.38 | 8.53 | 12.71 | 12.16 | 12.20 | 11.94 | 11.86 | 12.17 | 1.43 |
| #36 | 02_D07_44_04 | 6.75 | 6.94 | 6.63 | 6.96 | 6.59 | 6.77 | 11.38 | 10.46 | 11.48 | 11.49 | 11.37 | 11.23 | 1.66 |
| #37 | 02_D07_44_08 | 6.99 | 6.90 | 7.59 | 6.86 | 7.02 | 7.07 | 10.61 | 10.55 | 10.54 | 10.79 | 10.75 | 10.65 | 1.51 |
| #38 | 02_D07_44_09 | 7.15 | 6.66 | 6.91 | 7.41 | 7.85 | 7.20 | 10.96 | 10.68 | 11.65 | 10.60 | 11.53 | 11.08 | 1.54 |
| #39 | 02_D07_44_10 | 7.11 | 7.02 | 7.10 | 7.52 | 7.17 | 7.18 | 10.05 | 9.90 | 11.17 | 10.82 | 11.05 | 10.60 | 1.48 |
| #40 | 02_F04_26_01 | 5.92 | 6.50 | 6.07 | 5.47 | 5.53 | 5.90 | 10.68 | 10.58 | 10.37 | 9.91 | 10.33 | 10.37 | 1.76 |
| #41 | 02_F04_26_02 | 7.97 | 8.07 | 7.74 | 7.36 | 7.77 | 7.78 | 11.25 | 11.96 | 11.74 | 12.15 | 12.51 | 11.92 | 1.53 |
| #42 | 02_F04_26_04 | 6.92 | 7.66 | 7.22 | 7.33 | 7.50 | 7.32 | 9.48 | 10.70 | 10.53 | 10.78 | 10.69 | 10.43 | 1.42 |
| #43 | 02_F04_26_05 | 7.29 | 6.74 | 6.74 | 6.19 | 6.81 | 6.75 | 11.87 | 11.97 | 11.32 | 11.45 | 11.56 | 11.63 | 1.72 |
| #44 | 02_F09_29_01 | 8.04 | 7.86 | 7.34 | 7.45 | 7.85 | 7.71 | 13.36 | 13.53 | 13.31 | 12.58 | 13.15 | 13.19 | 1.71 |
| #45 | 02_F09_29_02 | 7.63 | 7.36 | 7.19 | 7.28 | 7.25 | 7.34 | 10.45 | 10.59 | 10.21 | 11.00 | 10.80 | 10.61 | 1.44 |
| #46 | 02_F09_29_04 | 6.31 | 7.28 | 7.40 | 7.06 | 7.05 | 7.02 | 11.37 | 11.80 | 11.80 | 11.37 | 11.51 | 11.57 | 1.65 |
| #47 | 02_F09_29_09 | 6.57 | 6.21 | 6.92 | 6.56 | 6.87 | 6.63 | 10.25 | 10.15 | 10.98 | 10.60 | 11.21 | 10.64 | 1.61 |
| #48 | 02_G01_52_06 | 7.71 | 8.74 | 8.18 | 8.00 | 8.27 | 8.18 | 12.23 | 12.19 | 12.24 | 12.65 | 12.24 | 12.31 | 1.51 |
| #49 | 02_G01_52_07 | 8.40 | 7.95 | 7.95 | 7.64 | 7.85 | 7.96 | 11.98 | 11.27 | 11.44 | 11.98 | 11.81 | 11.70 | 1.47 |
| #50 | 02_G01_52_09 | 8.56 | 8.51 | 9.03 | 8.83 | 8.82 | 8.75 | 13.25 | 12.73 | 13.33 | 13.87 | 13.82 | 13.40 | 1.53 |
| #52 | 02_G01_52_10 | 7.68 | 7.11 | 7.47 | 6.56 | 7.01 | 7.17 | 10.89 | 10.95 | 11.30 | 10.26 | 10.96 | 10.87 | 1.52 |
| #51 | 02_G01_52_10 | 8.66 | 8.53 | 8.11 | 8.09 | 8.54 | 8.39 | 14.08 | 14.20 | 14.61 | 14.81 | 14.12 | 14.36 | 1.71 |
| #53 | 02_G08_29_01 | 7.32 | 7.06 | 6.98 | 7.18 | 7.14 | 7.13 | 10.80 | 10.18 | 10.09 | 11.49 | 11.16 | 10.74 | 1.51 |
| #54 | 02_G08_29_02 | 7.61 | 7.17 | 7.64 | 7.13 | 7.46 | 7.40 | 11.44 | 10.02 | 10.87 | 11.84 | 10.79 | 10.99 | 1.48 |
| #55 | 02_G08_29_03 | 6.92 | 7.03 | 6.87 | 7.46 | 6.95 | 7.05 | 9.58 | 9.55 | 9.39 | 9.03 | 9.33 | 9.38 | 1.33 |
| #56 | 02_G08_29_04 | 7.01 | 6.66 | 7.19 | 6.72 | 7.13 | 6.94 | 11.86 | 11.51 | 11.20 | 10.66 | 11.18 | 11.28 | 1.62 |
| #57 | 02_G08_29_05 | 7.95 | 7.15 | 7.99 | 7.83 | 7.58 | 7.70 | 10.18 | 10.70 | 10.88 | 10.97 | 10.57 | 10.66 | 1.38 |
| #58 | 02_G08_29_08 | 7.77 | 7.08 | 7.47 | 6.63 | 7.15 | 7.22 | 11.89 | 10.95 | 11.30 | 10.26 | 10.96 | 11.07 | 1.53 |
| #59 | 02_G08_29_09 | 6.97 | 6.56 | 6.84 | 7.18 | 6.33 | 6.77 | 10.47 | 11.27 | 11.06 | 11.40 | 10.85 | 11.01 | 1.63 |
| #60 | 02_G08_29_10 | 6.97 | 6.91 | 6.55 | 6.73 | 6.81 | 6.79 | 10.27 | 9.93 | 10.44 | 10.00 | 9.73 | 10.07 | 1.48 |
| #61 | 03_C06_58_04 | 7.56 | 8.08 | 7.83 | 7.96 | 7.53 | 7.79 | 11.83 | 11.67 | 11.44 | 12.08 | 11.77 | 11.76 | 1.51 |
| #62 | 03_C06_58_11 | 6.75 | 6.94 | 6.63 | 6.55 | 6.59 | 6.69 | 11.38 | 10.62 | 11.48 | 11.49 | 11.37 | 11.27 | 1.68 |
| #63 | 03_C06_58_13 | 7.91 | 8.00 | 8.51 | 7.90 | 7.64 | 7.99 | 9.54 | 10.27 | 9.90 | 9.35 | 9.87 | 9.79 | 1.22 |
| #64 | 03_D03_52_09 | 7.54 | 7.81 | 7.97 | 7.82 | 7.75 | 7.78 | 10.94 | 10.28 | 10.96 | 11.57 | 10.70 | 10.89 | 1.40 |
| #65 | 04_D08_45_01 | 7.11 | 7.40 | 7.11 | 7.22 | 7.32 | 7.23 | 10.46 | 10.64 | 10.57 | 10.82 | 10.80 | 10.66 | 1.47 |
| #66 | 04_D08_45_01 | 8.25 | 8.37 | 8.31 | 8.16 | 8.14 | 8.25 | 13.04 | 13.25 | 13.68 | 13.19 | 12.97 | 13.23 | 1.60 |
| #67 | 04_E03_35_01 | 7.77 | 7.06 | 7.89 | 7.02 | 7.18 | 7.38 | 10.60 | 11.01 | 11.91 | 10.28 | 10.68 | 10.90 | 1.48 |
| #68 | 04_E03_35_05 | 7.48 | 7.38 | 7.53 | 7.60 | 7.40 | 7.48 | 10.52 | 10.32 | 10.52 | 10.56 | 10.34 | 10.45 | 1.40 |
| #69 | 04_E03_35_07 | 7.71 | 7.63 | 7.45 | 7.39 | 7.35 | 7.50 | 11.77 | 11.90 | 11.90 | 10.74 | 12.54 | 11.77 | 1.57 |
| #70 | 08_A11_24_01 | 8.38 | 8.21 | 7.92 | 8.68 | 8.33 | 8.30 | 13.63 | 13.55 | 13.11 | 14.62 | 13.25 | 13.63 | 1.64 |
| #71 | 08_A11_24_02 | 7.91 | 8.03 | 8.60 | 8.05 | 7.69 | 8.06 | 10.77 | 10.60 | 11.71 | 10.02 | 10.01 | 10.62 | 1.32 |
| #72 | 08_A11_24_02 | 7.71 | 8.63 | 8.45 | 8.39 | 7.35 | 8.10 | 10.77 | 11.90 | 12.90 | 10.74 | 11.54 | 11.57 | 1.43 |
| #73 | 08_A11_24_03 | 8.60 | 8.37 | 8.42 | 8.99 | 8.96 | 8.67 | 13.15 | 11.45 | 11.97 | 11.45 | 11.94 | 11.99 | 1.38 |
| #74 | 08_A11_24_04 | 7.11 | 7.02 | 7.10 | 7.52 | 7.17 | 7.18 | 10.46 | 9.99 | 11.17 | 10.82 | 11.05 | 10.70 | 1.49 |
| #75 | 08_A11_24_05 | 8.41 | 8.16 | 7.80 | 8.32 | 8.20 | 8.18 | 11.10 | 11.53 | 11.79 | 11.49 | 11.66 | 11.51 | 1.41 |
| #76 | 08_A11_24_06 | 8.07 | 7.95 | 8.50 | 8.79 | 7.67 | 8.20 | 11.93 | 12.04 | 12.03 | 11.75 | 11.36 | 11.82 | 1.44 |
| #77 | 08_A11_24_07 | 7.31 | 7.79 | 7.98 | 8.06 | 8.05 | 7.84 | 10.37 | 10.80 | 11.80 | 11.37 | 11.51 | 11.17 | 1.43 |
| #78 | 08_A11_24_08 | 8.28 | 8.16 | 7.92 | 8.22 | 8.56 | 8.23 | 12.92 | 11.73 | 11.69 | 11.88 | 12.84 | 12.21 | 1.48 |
| #79 | 08_A11_24_09 | 8.25 | 8.75 | 8.31 | 8.16 | 8.42 | 8.38 | 12.04 | 13.25 | 13.68 | 12.19 | 12.72 | 12.78 | 1.53 |
| #80 | 08_C02_25_10 | 6.51 | 7.62 | 6.90 | 7.17 | 7.60 | 7.16 | 11.41 | 11.04 | 11.00 | 11.28 | 10.20 | 10.99 | 1.53 |
| #81 | 08_C02_27_10 | 7.51 | 7.62 | 6.90 | 7.17 | 7.60 | 7.36 | 11.41 | 11.04 | 12.00 | 12.28 | 11.20 | 11.59 | 1.57 |
| #82 | 08_C10_27_09 | 6.43 | 6.78 | 6.33 | 6.90 | 7.19 | 6.73 | 11.40 | 10.82 | 10.81 | 11.37 | 10.83 | 11.04 | 1.64 |
| #83 | 08_G08_29_01 | 6.75 | 6.94 | 6.84 | 6.64 | 6.79 | 6.79 | 11.38 | 10.62 | 11.00 | 11.42 | 10.37 | 10.96 | 1.61 |
| #84 | 09_A11_12_01 | 7.55 | 7.78 | 6.60 | 7.68 | 7.48 | 7.42 | 10.18 | 10.53 | 10.78 | 10.38 | 10.61 | 10.50 | 1.42 |
| #85 | 09_A11_12_02 | 6.28 | 6.35 | 6.81 | 7.07 | 7.11 | 6.72 | 9.87 | 9.92 | 10.00 | 9.98 | 9.92 | 9.94 | 1.48 |
| #86 | 09_A11_12_03 | 8.42 | 7.85 | 7.37 | 8.36 | 8.53 | 8.11 | 11.81 | 11.57 | 11.72 | 11.66 | 11.63 | 11.68 | 1.44 |
| #87 | 09_A11_12_04 | 8.07 | 8.07 | 8.17 | 8.50 | 8.01 | 8.16 | 11.36 | 11.55 | 10.86 | 11.89 | 11.91 | 11.51 | 1.41 |
| #88 | 09_A11_12_05 | 7.69 | 7.90 | 8.01 | 7.85 | 7.94 | 7.88 | 12.76 | 13.37 | 12.43 | 12.93 | 12.81 | 12.86 | 1.63 |
| #89 | 09_A11_12_06 | 7.15 | 6.66 | 6.91 | 7.41 | 7.85 | 7.20 | 10.96 | 10.68 | 11.53 | 10.60 | 11.29 | 11.01 | 1.53 |
| #90 | 09_A11_12_07 | 7.53 | 7.48 | 7.43 | 7.30 | 7.19 | 7.39 | 10.40 | 10.82 | 10.81 | 10.37 | 10.83 | 10.64 | 1.44 |
| #91 | 09_A11_12_08 | 8.06 | 8.19 | 8.01 | 8.64 | 8.18 | 8.22 | 11.71 | 11.92 | 11.23 | 11.52 | 11.91 | 11.66 | 1.42 |
| #92 | 09_A11_12_09 | 6.43 | 6.78 | 6.33 | 6.90 | 7.19 | 6.73 | 9.40 | 9.82 | 9.81 | 9.37 | 9.83 | 9.64 | 1.43 |
| #93 | 09_G04_39_03 | 7.16 | 7.68 | 7.51 | 7.32 | 7.58 | 7.45 | 11.89 | 11.00 | 11.11 | 11.62 | 11.78 | 11.48 | 1.54 |
| #94 | 09_G04_39_04 | 7.65 | 7.38 | 7.64 | 7.59 | 7.72 | 7.59 | 11.55 | 11.94 | 11.64 | 12.09 | 12.03 | 11.85 | 1.56 |
| #95 | 09_G04_39_05 | 7.84 | 7.50 | 7.81 | 7.66 | 7.57 | 7.68 | 11.91 | 11.03 | 11.52 | 11.55 | 11.05 | 11.41 | 1.49 |
| #96 | 09_H10_31_10 | 7.61 | 7.17 | 7.64 | 7.13 | 7.46 | 7.40 | 11.44 | 10.02 | 10.87 | 11.84 | 10.79 | 10.99 | 1.48 |
| #97 | 09_H10_31_10 | 8.00 | 8.75 | 8.60 | 8.32 | 8.62 | 8.46 | 13.22 | 13.08 | 13.42 | 13.22 | 13.34 | 13.25 | 1.57 |
| #98 | 09_H11_12_02 | 6.94 | 7.04 | 6.64 | 6.81 | 6.85 | 6.85 | 9.31 | 9.87 | 9.77 | 9.65 | 9.43 | 9.61 | 1.40 |
| #99 | 09_H11_12_05 | 7.48 | 6.98 | 7.53 | 7.60 | 6.90 | 7.30 | 10.52 | 9.92 | 10.52 | 10.56 | 9.94 | 10.29 | 1.41 |
| #100 | 09_H11_12_06 | 7.20 | 7.75 | 7.60 | 7.32 | 7.62 | 7.50 | 11.22 | 11.71 | 11.42 | 11.82 | 11.83 | 11.60 | 1.55 |
| #101 | 09_H11_12_07 | 6.81 | 7.24 | 6.35 | 6.53 | 6.64 | 6.72 | 9.16 | 10.23 | 9.41 | 9.54 | 9.12 | 9.49 | 1.41 |
| #102 | 09_H11_12_10 | 7.14 | 7.43 | 7.49 | 7.98 | 7.19 | 7.45 | 10.61 | 10.65 | 11.04 | 11.20 | 11.07 | 10.91 | 1.47 |
| #103 | 09_H11_12_11 | 7.78 | 7.69 | 7.36 | 7.50 | 8.38 | 7.74 | 13.11 | 12.99 | 12.21 | 12.53 | 13.19 | 12.81 | 1.65 |
| #104 | 09_H11_12_13 | 8.51 | 8.26 | 7.86 | 8.32 | 8.20 | 8.23 | 12.20 | 12.00 | 12.16 | 12.66 | 11.92 | 12.19 | 1.48 |
| #105 | 09_H11_12_17 | 7.14 | 7.43 | 7.49 | 7.98 | 7.19 | 7.45 | 10.61 | 10.65 | 10.04 | 10.20 | 10.07 | 10.31 | 1.38 |

**Supplementary Table 2.** Summary statistics of Illumina whole-genome sequencing data for the parental lines, NHN and Pink+4 (#20A09), and the high-CRE and low-CRE bulks.

| **Sample** | **Raw**  **Reads**  **(Million)** | **Clean**  **Read**  **(Million)** | **Clean**  **Base**  **(Gb)** | **Alignment**  **(%)** | **Genome**  **Coverage**  **(%)** | **Average**  **Depth**  **(×)** |
| --- | --- | --- | --- | --- | --- | --- |
| NHN | 63.88 | 56.90 | 8.53 | 0.88 | 94.81 | 19.81 |
| Pink+4 (#20A09) | 106.09 | 80.98 | 12.05 | 0.81 | 90.17 | 25.65 |
| Low-CRE bulk | 66.23 | 50.83 | 7.54 | 0.76 | 95.89 | 15.18 |
| High-CRE bulk | 63.06 | 48.40 | 7.18 | 0.83 | 95.54 | 15.84 |

**Supplementary Table 3.** Distribution of single nucleotide polymorphisms (SNPs) and insertion/deletion polymorphisms (InDels) identified across the 12 rice chromosomes between the high-CRE and low-CRE.

| **Chromosome** | **Length** | **Total number of variants**  **(depth ≥ 3)** | **Total number of variants**  **(filtered, depth ≥ 18)** |
| --- | --- | --- | --- |
| 1 | 43,270,923 | 141,855 | 2,772 |
| 2 | 35,937,250 | 107,559 | 2,211 |
| 3 | 36,413,819 | 135,064 | 2,187 |
| 4 | 35,502,694 | 111,209 | 1,472 |
| 5 | 29,958,434 | 91,405 | 1,560 |
| 6 | 31,248,787 | 83,092 | 1,521 |
| 7 | 29,697,621 | 94,263 | 1,506 |
| 8 | 28,443,022 | 102,486 | 1,306 |
| 9 | 23,012,720 | 79,621 | 1,266 |
| 10 | 23,207,287 | 97,929 | 1,032 |
| 11 | 29,021,106 | 105,824 | 953 |
| 12 | 27,531,856 | 84,315 | 988 |
| **Total** | **373,245,519** | **1,234,622** | **18,774** |

**Supplementary Table 4.** List of predicted 104 candidate genes and genomic intervals in the *qCRE1*, *qCRE4*, *qCRE7*, *qCRE10.1*, *qCRE10.2* and *qCRE12* regions.

| **No.** | **Locus ID** | **Description** | **Position** |
| --- | --- | --- | --- |
| 1 | Os01g0706332 | Hypothetical protein. | chr01:29298120..29299495(- strand) |
| 2 | Os01g0706400 | Protein of unknown function DUF292 family protein. | chr01:29306448..29312511(+ strand) |
| 3 | Os01g0706700 | Protein of unknown function DUF590 family protein. | chr01:29318195..29322559(- strand) |
| 4 | Os01g0706850 | Non-protein coding transcript. | chr01:29326816..29327360(+ strand) |
| 5 | Os01g0708300 | Protein of unknown function DUF803 domain containing protein. | chr01:29423549..29427813(+ strand) |
| 6 | Os01g0708400 | Non-protein coding transcript. | chr01:29436121..29436450(+ strand) |
| 7 | Os01g0708900 | Mitochondrial substrate carrier family protein. | chr01:29458644..29462865(+ strand) |
| 8 | Os01g0709000 | R2R3-MYB transcription factor 13. | chr01:29469565..29471507(+ strand) |
| 9 | Os01g0709750 | Hypothetical gene. | chr01:29484876..29488093(+ strand) |
| 10 | Os01g0710100 | Hypothetical conserved gene. | chr01:29508034..29509147(+ strand) |
| 11 | Os01g0711200 | LRR-receptor-like kinase (LRR-RLK) family protein. | chr01:29551666..29553996(+ strand) |
| 12 | Os01g0714100 | Conserved hypothetical protein. | chr01:29679124..29680754(- strand) |
| 13 | Os01g0715000 | Conserved hypothetical protein. | chr01:29736321..29739485(- strand) |
| 14 | Os01g0715600 | Similar to auxin efflux carrier component. | chr01:29775983..29778839(- strand) |
| 15 | Os01g0715900 | Pentatricopeptide repeat domain containing protein. | chr01:29786136..29788237(- strand) |
| 16 | Os01g0716200 | IQ calmodulin-binding region domain containing protein. | chr01:29801081..29806870(- strand) |
| 17 | Os01g0716300 | Conserved hypothetical protein. | chr01:29810721..29812563(- strand) |
| 18 | Os01g0716800 | Endonuclease/exonuclease/phosphatase domain containing protein. | chr01:29830265..29836090(+ strand) |
| 19 | Os01g0717400 | EGF-like, alliinase domain containing protein. | chr01:29874486..29877459(+ strand) |
| 20 | Os01g0718500 | Protein of unknown function DUF1070 domain containing protein. | chr01:29937455..29938094(+ strand) |
| 21 | Os01g0719100 | Similar to RING finger and CHY zinc finger domain-containing protein 1. | chr01:29965312..29970765(- strand) |
| 22 | Os01g0719300 | Sulfate transporter protein. | chr01:29987911..29994075(+ strand) |
| 23 | Os01g0720600 | Similar to Starch synthase IV. | chr01:30032428..30041425(- strand) |
| 24 | Os01g0720800 | Hypothetical conserved gene. | chr01:30048381..30049982(+ strand) |
| 25 | Os01g0720801 | Hypothetical gene. | chr01:30055456..30057472(- strand) |
| 26 | Os01g0721300 | Similar to cDNA clone:J013002N02, full insert sequence. | chr01:30075624..30077126(+ strand) |
| 27 | Os01g0722425 | Hypothetical protein. | chr01:30117870..30118492(- strand) |
| 28 | Os01g0723800 | Similar to P-glycoprotein homologue. | chr01:30189384..30195340(- strand) |
| 29 | Os04g0127900 | Conserved hypothetical protein. | chr04:1760660..1762304(+ strand) |
| 30 | Os04g0129350 | Hypothetical gene. | chr04:1844672..1846498(- strand) |
| 31 | Os04g0227500 | DSBA oxidoreductase family protein. | chr04:8528512..8533136(+ strand) |
| 32 | Os04g0228000 | Peptidase A1 domain containing protein. | chr04:8578980..8585626(+ strand) |
| 33 | Os04g0228600 | Similar to H0209A05.7 protein. | chr04:8614296..8616079(- strand) |
| 34 | Os07g0541800 | Similar to KI domain interacting kinase 1. | chr07:21407942..21411687(- strand) |
| 35 | Os07g0543100 | Similar to Beta-amylase. | chr07:21470797..21473327(+ strand) |
| 36 | Os07g0543300 | Similar to Beta-amylase. | chr07:21505640..21506607(+ strand) |
| 37 | Os07g0545500 | Similar to VMP4 protein. | chr07:21594798..21600723(- strand) |
| 38 | Os07g0545900 | Similar to EMB1417. | chr07:21621426..21624421(+ strand) |
| 39 | Os07g0546150 | Hypothetical protein. | chr07:21629498..21633448(- strand) |
| 40 | Os07g0548500 | Conserved hypothetical protein. | chr07:21760605..21764014(+ strand) |
| 41 | Os07g0549800 | Similar to RGP-3 (Fragment). | chr07:21823012..21825223(+ strand) |
| 42 | Os07g0549900 | Similar to Histone H4. | chr07:21827202..21827703(+ strand) |
| 43 | Os07g0550500 | S-Domain receptor like kinase-39. | chr07:21846328..21851114(- strand) |
| 44 | Os10g0142301 | Non-protein coding transcript. | chr10:2572703..2573795(+ strand) |
| 45 | Os10g0184432 | Non-protein coding transcript. | chr10:5799171..5799390(+ strand) |
| 46 | Os10g0184871 | Similar to Protein kinase domain containing protein. | chr10:5826151..5827881(+ strand) |
| 47 | Os10g0192301 | Non-protein coding transcript. | chr10:6416709..6417205(+ strand) |
| 48 | Os10g0193100 | Conserved hypothetical protein. | chr10:6484463..6486741(+ strand) |
| 49 | Os10g0200800 | Similar to Phosphatidylinositol glycan synthesis class F protein. | chr10:7121108..7141151(- strand) |
| 50 | Os10g0202200 | Hypothetical conserved gene. | chr10:7270047..7270697(- strand) |
| 51 | Os10g0202350 | Similar to Seven transmembrane-spanning protein. | chr10:7271466..7271912(+ strand) |
| 52 | Os10g0202501 | Hypothetical gene. | chr10:7301658..7304913(- strand) |
| 53 | Os10g0203100 | Hypothetical conserved gene. | chr10:7341903..7342367(- strand) |
| 54 | Os10g0203600 | DNA-binding SAP domain containing protein. | chr10:7385766..7395013(+ strand) |
| 55 | Os10g0320400 | Similar to ATP synthase gamma chain. | chr10:8691901..8697228(+ strand) |
| 56 | Os10g0321700 | Similar to Ribonucleoprotein like protein. | chr10:8786980..8796460(- strand) |
| 57 | Os10g0322300 | Conserved hypothetical protein. | chr10:8837124..8838148(- strand) |
| 58 | Os10g0322600 | Hypothetical gene. | chr10:8846840..8847352(+ strand) |
| 59 | Os10g0323000 | Conserved hypothetical protein. | chr10:8891489..8910642(+ strand) |
| 60 | Os10g0323500 | Similar to Beta-glucosidase. | chr10:8920265..8926170(+ strand) |
| 61 | Os10g0323900 | Similar to Profilin A. | chr10:8937202..8938316(- strand) |
| 62 | Os10g0324600 | Zinc finger, C2H2-like domain containing protein. | chr10:8980283..8985013(+ strand) |
| 63 | Os10g0324900 | Homolog of Arabidopsis LHP1. | chr10:8992297..8996949(+ strand) |
| 64 | Os10g0325400 | Remorin, C-terminal domain containing protein. | chr10:9000705..9002772(+ strand) |
| 65 | Os10g0328100 | Conserved hypothetical protein. | chr10:9170819..9171265(+ strand) |
| 66 | Os10g0328600 | Hypothetical protein. | chr10:9212944..9216458(- strand) |
| 67 | Os10g0328700 | Tetratricopeptide-like helical domain containing protein. | chr10:9213110..9215332(+ strand) |
| 68 | Os10g0329900 | Similar to Protein kinase domain containing protein. | chr10:9284772..9285986(- strand) |
| 69 | Os10g0339290 | Similar to Alpha-1,2-fucosidase. | chr10:9861144..9868032(- strand) |
| 70 | Os10g0339400 | IQ calmodulin-binding region domain containing protein. | chr10:9910689..9917699(+ strand) |
| 71 | Os10g0339650 | Non-protein coding transcript. | chr10:9930896..9931451(+ strand) |
| 72 | Os10g0340100 | Conserved hypothetical protein. | chr10:9974284..9976309(+ strand) |
| 73 | Os10g0340301 | Conserved hypothetical protein. | chr10:9984427..9987827(+ strand) |
| 74 | Os10g0343050 | Non-protein coding transcript. | chr10:10167156..10167569(- strand) |
| 75 | Os10g0343900 | Thioredoxin fold domain containing protein. | chr10:10216071..10218319(- strand) |
| 76 | Os10g0344550 | Conserved hypothetical protein. | chr10:10257767..10258401(+ strand) |
| 77 | Os10g0347800 | Similar to Formin-like protein 4. | chr10:10482035..10486120(- strand) |
| 78 | Os10g0358700 | Pentatricopeptide repeat domain containing protein. | chr10:10991287..10997385(- strand) |
| 79 | Os10g0360800 | LRR-receptor-like kinase (LRR-RLK) family protein. | chr10:11088171..11091811(+ strand) |
| 80 | Os10g0363100 | Conserved hypothetical protein. | chr10:11250926..11255580(+ strand) |
| 81 | Os10g0363300 | Acetyl-CoA carboxylase. | chr10:11273893..11284909(+ strand) |
| 82 | Os12g0542200 | Conserved hypothetical protein. | chr12:21711496..21713941(- strand) |
| 83 | Os12g0542300 | Conserved hypothetical protein. | chr12:21766760..21767380(+ strand) |
| 84 | Os12g0542601 | Conserved hypothetical protein. | chr12:21783684..21786014(- strand) |
| 85 | Os12g0543650 | Hypothetical gene. | chr12:21869759..21871355(- strand) |
| 86 | Os12g0545600 | Conserved hypothetical protein. | chr12:22022000..22031120(+ strand) |
| 87 | Os12g0545900 | Conserved hypothetical protein. | chr12:22047697..22055332(+ strand) |
| 88 | Os12g0546800 | Alpha expansin 26. | chr12:22089434..22091864(+ strand) |
| 89 | Os12g0547500 | Kinesin-14 family member. | chr12:22126680..22133291(+ strand) |
| 90 | Os12g0548200 | Hypothetical conserved gene. | chr12:22173286..22180480(- strand) |
| 91 | Os12g0550600 | Hypothetical protein. | chr12:22283176..22284740(- strand) |
| 92 | Os12g0556100 | Similar to fiber protein Fb2. | chr12:22616804..22622177(+ strand) |
| 93 | Os12g0556500 | Calmodulin binding protein-like family protein. | chr12:22648120..22656258(+ strand) |
| 94 | Os12g0557800 | Pentatricopeptide repeat domain containing protein. | chr12:22723146..22726841(- strand) |
| 95 | Os12g0558133 | Similar to SPA4. | chr12:22736219..22736862(+ strand) |
| 96 | Os12g0558366 | Similar to F-box domain containing protein. | chr12:22773516..22774906(+ strand) |
| 97 | Os12g0560300 | Small GTPase. | chr12:22939792..22943689(- strand) |
| 98 | Os12g0560500 | Pseudouridine synthase domain containing protein. | chr12:22948747..22951075(- strand) |
| 99 | Os12g0560700 | Similar to PROLIFERA protein. | chr12:22954078..22959404(- strand) |
| 100 | Os12g0561000 | Cytochrome c oxidase polypeptide Vc. | chr12:22969436..22972239(- strand) |
| 101 | Os12g0561750 | Similar to ferroportin protein family. | chr12:23009303..23012933(- strand) |
| 102 | Os12g0609800 | WD40/YVTN repeat-like domain containing protein. | chr12:25752130..25758238(+ strand) |
| 103 | Os12g0610500 | SAM binding motif domain containing protein. | chr12:25790709..25796263(+ strand) |
| 104 | Os12g0611100 | Similar to serine/threonine-protein kinase receptor. | chr12:25820882..25824699(- strand) |

**Supplementary Table 5.** SNP positions exhibiting high Δ(SNP-index) values within the candidate genomic region are indicated. Bold, italicised, and underlined text denotes the selected KASP markers, for which ΔSNP indexes ≥ 0.5, for single-marker analysis in the BC_1_F_2_ mapping population.

| **No.** | **CHR.** | **POSI** | **bulk1_SNPindex** | **bulk2_SNPindex** | Δ**SNPindex** | **REF** | **ALT** | **Gene ID** | **Description** |
| --- | --- | --- | --- | --- | --- | --- | --- | --- | --- |
| 1 | 1 | 29302064 | 0.4211 | 0.8571 | 0.4361 | A | C | Os01g0706332 | Hypothetical protein. |
| 2 | 1 | 29307528 | 0.4 | 0.8846 | 0.4846 | A | C | Os01g0706400 | Protein of unknown function DUF292 domain containing protein. |
| ***3*** | ***1*** | ***29312181*** | ***0.4*** | ***0.931*** | ***0.531*** | ***T*** | ***C*** | ***Os01g0706400*** | ***Protein of unknown function DUF292 domain containing protein.*** |
| ***4*** | ***1*** | ***29322283*** | ***0.381*** | ***0.913*** | ***0.5321*** | ***C*** | ***T*** | ***Os01g0706700*** | ***Protein of unknown function DUF803 domain containing protein.*** |
| ***5*** | ***1*** | ***29324976*** | ***0.3333*** | ***0.8947*** | ***0.5614*** | ***G*** | ***A*** | ***Os01g0706700*** | ***Protein of unknown function DUF803 domain containing protein.*** |
| 6 | 1 | 29330608 | 0.4 | 0.7895 | 0.3895 | C | T | Os01g0706850 | Non-protein coding transcript. |
| 7 | 1 | 29429194 | 0.3889 | 0.7778 | 0.3889 | C | T | Os01g0708300 | Protein of unknown function DUF803 domain containing protein. |
| 8 | 1 | 29433702 | 0.4762 | 0.8421 | 0.3659 | A | G | Os01g0708400 | Non-protein coding transcript. |
| 9 | 1 | 29464488 | 0.4444 | 0.8571 | 0.4127 | C | T | Os01g0708900 | Mitochondrial substrate carrier family protein. |
| 10 | 1 | 29468938 | 0.6111 | 1 | 0.3889 | C | T | Os01g0709000 | R2R3-MYB transcription factor 13 |
| 11 | 1 | 29481271 | 0.4091 | 0.8462 | 0.4371 | T | A | Os01g0709750 | Hypothetical gene. |
| 12 | 1 | 29503938 | 0.6111 | 1 | 0.3889 | T | C | Os01g0710100 | Hypothetical conserved gene. |
| 13 | 1 | 29547284 | 0.3684 | 0.8333 | 0.4649 | G | A | Os01g0711200 | LRR-receptor-like kinase (LRR-RLK) family protein |
| 14 | 1 | 29681007 | 0.4737 | 0.8636 | 0.39 | T | C | Os01g0714100 | Conserved hypothetical protein. |
| ***15*** | ***1*** | ***29733026*** | ***0.35*** | ***0.9524*** | ***0.6024*** | ***C*** | ***T*** | ***Os01g0715000*** | ***Conserved hypothetical protein.*** |
| 16 | 1 | 29771708 | 0.3889 | 0.8421 | 0.4532 | G | A | Os01g0715600 | Similar to auxin efflux carrier component. |
| ***17*** | ***1*** | ***29771713*** | ***0.3684*** | ***0.8889*** | ***0.5205*** | ***C*** | ***T*** | ***Os01g0715600*** | ***Similar to auxin efflux carrier component.*** |
| 18 | 1 | 29791484 | 0.35 | 0.8889 | 0.5389 | A | G | Os01g0715900 | Pentatricopeptide repeat domain containing protein. |
| 19 | 1 | 29791725 | 0.3889 | 0.8261 | 0.4372 | T | C | Os01g0715900 | Pentatricopeptide repeat domain containing protein. |
| 20 | 1 | 29797420 | 0.3889 | 0.75 | 0.3611 | T | C | Os01g0716200 | Calmodulin-binding region domain containing protein. |
| 21 | 1 | 29798361 | 0.4783 | 0.88 | 0.4017 | C | T | Os01g0716200 | Calmodulin-binding region domain containing protein. |
| 22 | 1 | 29799568 | 0.45 | 0.9048 | 0.4548 | T | C | Os01g0716200 | Calmodulin-binding region domain containing protein. |
| 23 | 1 | 29804187 | 0.4737 | 0.8333 | 0.3596 | A | G | Os01g0716200 | Calmodulin-binding region domain containing protein. |
| 24 | 1 | 29806316 | 0.6 | 0.9655 | 0.3655 | C | T | Os01g0716300 | Conserved hypothetical protein. |
| 25 | 1 | 29831091 | 0.4815 | 0.8333 | 0.3519 | A | G | Os01g0716800 | Endonuclease/exonuclease/phosphatase domain containing protein. |
| 26 | 1 | 29873310 | 0.48 | 0.8261 | 0.3461 | A | T | Os01g0717400 | EGF-like, alliinase domain containing protein. |
| 27 | 1 | 29938080 | 0.4737 | 0.9444 | 0.4708 | A | G | Os01g0718500 | Protein of unknown function DUF1070 domain containing protein. |
| **28** | **1** | **29975201** | **0.4286** | **0.9444** | **0.5159** | **A** | **G** | **Os01g0719100** | **RING finger and CHY zinc finger domain-containing protein 1.** |
| 29 | 1 | 29991484 | 0.4211 | 0.9048 | 0.4837 | T | C | Os01g0719300 | Sulfate transporter protein. |
| ***30*** | ***1*** | ***30038156*** | ***0.3684*** | ***0.8889*** | ***0.5205*** | ***A*** | ***G*** | ***Os01g0720600*** | ***Similar to Starch synthase IV.*** |
| 31 | 1 | 30048939 | 0.3462 | 0.7619 | 0.4158 | C | T | Os01g0720800 | Hypothetical conserved gene. |
| ***32*** | ***1*** | ***30057841*** | ***0.3333*** | ***0.9*** | ***0.5667*** | ***C*** | ***T*** | ***Os01g0720801*** | ***Hypothetical gene.*** |
| 33 | 1 | 30077000 | 0.5238 | 0.9444 | 0.4206 | C | T | Os01g0721300 | Similar to cDNA clone:J013002N02, full insert sequence. |
| 34 | 1 | 30119594 | 0.4211 | 0.7778 | 0.3567 | T | C | Os01g0722425 | Hypothetical protein. |
| ***35*** | ***1*** | ***30196202*** | ***0.4*** | ***0.9444*** | ***0.5444*** | ***T*** | ***C*** | ***Os01g0723800*** | ***Similar to P-glycoprotein homologue.*** |
| 36 | 4 | 1760915 | 0.8947 | 0.4762 | -0.4185 | A | T | Os04g0127900 | Conserved hypothetical protein. |
| ***37*** | ***4*** | ***1849181*** | ***0.9565*** | ***0.3636*** | ***-0.5929*** | ***C*** | ***T*** | ***Os04g0129350*** | ***Hypothetical gene.*** |
| 38 | 4 | 8532885 | 0.9474 | 0.4762 | -0.4712 | G | A | Os04g0227500 | DSBA oxidoreductase family protein. |
| 39 | 4 | 8585299 | 0.85 | 0.381 | -0.469 | T | G | Os04g0228000 | Peptidase A1 domain containing protein. |
| 40 | 4 | 8615859 | 0.85 | 0.4348 | -0.4152 | T | C | Os04g0228600 | Similar to H0209A05.7 protein. |
| ***41*** | ***7*** | ***21413833*** | ***0.25*** | ***0.8333*** | ***0.5833*** | ***G*** | ***A*** | ***Os07g0541800*** | ***Similar to KI domain interacting kinase 1.*** |
| 42 | 7 | 21414008 | 0.2963 | 0.7391 | 0.4428 | C | T | Os07g0541800 | Similar to KI domain interacting kinase 1. |
| 43 | 7 | 21478825 | 0.3684 | 0.7895 | 0.4211 | A | G | Os07g0543100 | Similar to Beta-amylase. |
| 44 | 7 | 21597157 | 0.3158 | 0.7619 | 0.4461 | C | T | Os07g0545500 | Similar to VMP4 protein. |
| ***45*** | ***7*** | ***21599574*** | ***0.3158*** | ***0.9048*** | ***0.589*** | ***A*** | ***G*** | ***Os07g0545500*** | ***Similar to VMP4 protein.*** |
| 46 | 7 | 21621489 | 0.4211 | 0.9231 | 0.502 | C | T | Os07g0545900 | Similar to EMB1417. |
| 47 | 7 | 21637433 | 0.3636 | 0.8 | 0.4364 | A | G | Os07g0546150 | Hypothetical protein. |
| 48 | 7 | 21761158 | 0.25 | 0.7308 | 0.4808 | A | G | Os07g0548500 | Conserved hypothetical protein. |
| 49 | 7 | 21821321 | 0.5 | 0.8889 | 0.3889 | T | C | Os07g0549800 | Similar to RGP-3 (Fragment). |
| 50 | 7 | 21825926 | 0.381 | 0.8696 | 0.4886 | C | T | Os07g0549900 | Similar to Histone H4. |
| 51 | 7 | 21844258 | 0.4737 | 0.8889 | 0.4152 | A | G | Os07g0550500 | S-Domain receptor like kinase-39, Response to R. solani |
| 52 | 10 | 2569656 | 0.3636 | 0.7 | 0.3364 | G | A | Os10g0142301 | Non-protein coding transcript. |
| 53 | 10 | 5809039 | 0.55 | 0.9 | 0.35 | A | G | Os10g0184432 | Non-protein coding transcript. |
| 54 | 10 | 6425688 | 0.2381 | 0.7083 | 0.4702 | C | T | Os10g0192301 | Non-protein coding transcript. |
| 55 | 10 | 7251465 | 0.35 | 0.7778 | 0.4278 | A | G | Os10g0200800 | Phosphatidylinositol glycan synthesis class F protein. |
| 56 | 10 | 7277247 | 0.3929 | 0.8261 | 0.4332 | T | A | Os10g0202350 | Seven transmembrane-spanning protein. |
| ***57*** | ***10*** | ***7303735*** | ***0.3684*** | ***0.88*** | ***0.5116*** | ***T*** | ***A*** | ***Os10g0202501*** | ***Hypothetical gene.*** |
| 58 | 10 | 7358424 | 0.3333 | 0 | -0.3333 | T | C | Os10g0203100 | Hypothetical conserved gene. |
| 59 | 10 | 7369822 | 0.4091 | 0.75 | 0.3409 | C | T | Os10g0203100 | Hypothetical conserved gene. |
| 60 | 10 | 8702972 | 0.45 | 0.875 | 0.425 | A | G | Os10g0320400 | ATP synthase gamma chain, mitochondrial precursor. |
| 61 | 10 | 8764992 | 0.35 | 0.7 | 0.35 | G | A | Os10g0320400 | ATP synthase gamma chain, mitochondrial precursor. |
| ***62*** | ***10*** | ***8803290*** | ***0.1111*** | ***0.7619*** | ***0.6508*** | ***C*** | ***T*** | ***Os10g0321700*** | ***Similar to Ribonucleoprotein like protein.*** |
| 63 | 10 | 8835199 | 0.3478 | 0.7778 | 0.43 | G | A | Os10g0322300 | Conserved hypothetical protein. |
| ***64*** | ***10*** | ***8869299*** | ***0.2222*** | ***0.7619*** | ***0.5397*** | ***A*** | ***G*** | ***Os10g0322600*** | ***Hypothetical gene.*** |
| 65 | 10 | 8882589 | 0.2857 | 0.7727 | 0.487 | C | T | Os10g0322600 | Hypothetical gene. |
| 66 | 10 | 8917302 | 0.2222 | 0.7083 | 0.4861 | G | A | Os10g0323500 | Similar to Beta-glucosidase. |
| ***67*** | ***10*** | ***8935229*** | ***0.2143*** | ***0.8571*** | ***0.6429*** | ***C*** | ***T*** | ***Os10g0323900*** | ***Similar to Profilin A.*** |
| ***68*** | ***10*** | ***8984630*** | ***0.2222*** | ***0.8421*** | ***0.6199*** | ***G*** | ***A*** | ***Os10g0324600*** | ***Zinc finger, C_2_H_2_-like domain containing protein.*** |
| 69 | 10 | 8987888 | 0.5238 | 0.8889 | 0.3651 | G | C | Os10g0324900 | Homolog of Arabidopsis LHP1 |
| 70 | 10 | 9001933 | 0.2222 | 0.7143 | 0.4921 | G | A | Os10g0325400 | Remorin, C-terminal domain containing protein. |
| ***71*** | ***10*** | ***9195457*** | ***0.2632*** | ***0.7778*** | ***0.5146*** | ***G*** | ***C*** | ***Os10g0328100*** | ***Conserved hypothetical protein.*** |
| 72 | 10 | 9214622 | 0.44 | 0.7895 | 0.3495 | C | T | Os10g0328700 | Tetratricopeptide-like helical domain containing protein. |
| 73 | 10 | 9286812 | 0.1667 | 0.56 | 0.3933 | A | G | Os10g0329900 | Similar to Protein kinase domain containing protein. |
| 74 | 10 | 9287612 | 0.1429 | 0.6 | 0.4571 | T | C | Os10g0329900 | Similar to Protein kinase domain containing protein. |
| 75 | 10 | 9872354 | 0.0417 | 0.4 | 0.3583 | G | T | Os10g0339290 | Similar to Alpha-1,2-fucosidase. |
| ***76*** | ***10*** | ***9902249*** | ***0.3889*** | ***0.8947*** | ***0.5058*** | ***A*** | ***G*** | ***Os10g0339290*** | ***Similar to Alpha-1,2-fucosidase.*** |
| 77 | 10 | 9941322 | 0.4211 | 0.8696 | 0.4485 | T | C | Os10g0339650 | Non-protein coding transcript. |
| 78 | 10 | 9949725 | 0.35 | 0.7778 | 0.4278 | T | A | Os10g0339650 | Non-protein coding transcript. |
| 79 | 10 | 9984738 | 0.3684 | 0.8125 | 0.4441 | G | C | Os10g0340301 | Conserved hypothetical protein. |
| 80 | 10 | 10167890 | 0.45 | 0.8421 | 0.3921 | C | T | Os10g0343050 | Non-protein coding transcript. |
| ***81*** | ***10*** | ***10217652*** | ***0.0556*** | ***0.72*** | ***0.6644*** | ***A*** | ***T*** | ***Os10g0343900*** | ***Thioredoxin fold domain containing protein.*** |
| 82 | 10 | 10259042 | 0.4 | 0.7619 | 0.3619 | G | A | Os10g0344550 | Conserved hypothetical protein. |
| 83 | 10 | 10482896 | 0.5263 | 0.8947 | 0.3684 | T | G | Os10g0347800 | Similar to Formin-like protein 4. |
| 84 | 10 | 10993489 | 0.35 | 0.8421 | 0.4921 | G | A | Os10g0358700 | Pentatricopeptide repeat domain containing protein. |
| ***85*** | ***10*** | ***11089916*** | ***0.1905*** | ***0.9474*** | ***0.7569*** | ***A*** | ***G*** | ***Os10g0360800*** | ***LRR-receptor-like kinase (LRR-RLK) family protein.*** |
| ***86*** | ***10*** | ***11255488*** | ***0.2222*** | ***0.8571*** | ***0.6349*** | ***A*** | ***G*** | ***Os10g0363100*** | ***Similar to glycosyltransferase, family GT8.*** |
| ***87*** | ***10*** | ***11259388*** | ***0.1667*** | ***0.9565*** | ***0.7899*** | ***T*** | ***C*** | ***Os10g0363100*** | ***Similar to glycosyltransferase, family GT8.*** |
| 88 | 10 | 11262515 | 0.4737 | 0.85 | 0.3763 | T | G | Os10g0363100 | Similar to glycosyltransferase, family GT8. |
| ***89*** | ***10*** | ***11274471*** | ***0.2105*** | ***0.8333*** | ***0.6228*** | ***A*** | ***T*** | ***Os10g0363300*** | ***Acetyl-CoA carboxylase.*** |
| 90 | 12 | 21740759 | 0.5 | 0.8889 | 0.3889 | G | T | Os12g0542200 | Conserved hypothetical protein. |
| 91 | 12 | 21772922 | 0.55 | 0.9444 | 0.3944 | T | G | Os12g0542300 | Conserved hypothetical protein. |
| 92 | 12 | 21872694 | 0.4737 | 0.9444 | 0.4708 | G | A | Os12g0543650 | Hypothetical gene. |
| ***93*** | ***12*** | ***22020155*** | ***0.5*** | ***1*** | ***0.5*** | ***C*** | ***T*** | ***Os12g0545600*** | ***Conserved hypothetical protein.*** |
| 94 | 12 | 22021109 | 0.55 | 0.8947 | 0.3447 | C | T | Os12g0545600 | Conserved hypothetical protein. |
| 95 | 12 | 22070104 | 0.5556 | 0.9474 | 0.3918 | G | A | Os12g0545900 | Conserved hypothetical protein. |
| 96 | 12 | 22124236 | 0.5263 | 1 | 0.4737 | G | C | Os12g0547500 | Kinesin-14 family member, Non-processive. |
| 97 | 12 | 22124394 | 0.6111 | 1 | 0.3889 | G | A | Os12g0547500 | Kinesin-14 family member, Non-processive. |
| 98 | 12 | 22174777 | 0.3846 | 0.8571 | 0.4725 | C | T | Os12g0548200 | Hypothetical conserved gene. |
| 99 | 12 | 22286826 | 0.5909 | 1 | 0.4091 | G | A | Os12g0550600 | Hypothetical protein. |
| 100 | 12 | 22617445 | 0.5172 | 0.9474 | 0.4301 | C | G | Os12g0556100 | Similar to fiber protein Fb2. |
| 101 | 12 | 22660162 | 0.4737 | 0.9444 | 0.4708 | A | G | Os12g0556500 | Calmodulin binding protein-like family protein. |
| 102 | 12 | 22731435 | 0.4091 | 0.8333 | 0.4242 | T | A | Os12g0557800 | Pentatricopeptide repeat domain containing protein. |
| 103 | 12 | 22742907 | 0.6 | 1 | 0.4 | G | A | Os12g0558133 | Similar to SPA4; protein binding / signal transducer. |
| 104 | 12 | 22946287 | 0.4286 | 0.8421 | 0.4135 | C | A | Os12g0560300 | Small GTPase, Rice storage proteins proglutelin. |
| 105 | 12 | 22950785 | 0.48 | 0.9091 | 0.4291 | C | A | Os12g0560500 | Pseudouridine synthase domain containing protein. |
| 106 | 12 | 22955155 | 0.5 | 0.9167 | 0.4167 | T | C | Os12g0560700 | Similar to PROLIFERA protein. |
| 107 | 12 | 22976301 | 0.3889 | 0.9474 | 0.5585 | T | C | Os12g0561000 | Cytochrome c oxidase polypeptide Vc. |
| ***108*** | ***12*** | ***23010774*** | ***0.45*** | ***0.9545*** | ***0.5045*** | ***T*** | ***G*** | ***Os12g0561750*** | ***Similar to ferroportin protein family.*** |
| 109 | 12 | 25754133 | 0.4737 | 0.85 | 0.3763 | A | G | Os12g0609800 | WD40/YVTN repeat-like domain containing protein. |
| 110 | 12 | 25794081 | 0.4348 | 0.9355 | 0.5007 | T | A | Os12g0610500 | SAM binding motif domain containing protein. |
| 111 | 12 | 25825863 | 0.4 | 0.84 | 0.44 | C | T | Os12g0611100 | Similar to serine/threonine-protein kinase receptor. |

**Supplementary Table 6.** Sequences 5’->3’ of KASP markers used in this study.

| **No.** | **Name** | **Chr.** | **Position** | **Ref allele** | **Alt_allele** | **Primer FAM allele** | **Primer Hex_allele** | **Primer_common** |
| --- | --- | --- | --- | --- | --- | --- | --- | --- |
| 1 | >R0129312181 | 1 | 29312181 | T | C | TCCGACAGACTATGATTCGTTT | CTTCCGACAGACTATGATTCGTTC | GGCGATTGGTCCGAAGAGACATAAA |
| 2 | >R0129322283 | 1 | 29322283 | T | C | CCCTACGTCCTCCAGCTCCT | CCTACGTCCTCCAGCTCCC | ACTGCGTGGCGCGGCTGGT |
| 3 | >R0129324976 | 1 | 29324976 | A | G | ACTGACGAGCTGATGAGGAATCAA | CTGACGAGCTGATGAGGAATCAG | CACAAAGCAACAGATACGACAAGAGTTT |
| 4 | >R0129733026 | 1 | 29733026 | T | C | GAACTGGAAGATCAATTTGAGATCTTCA | AACTGGAAGATCAATTTGAGATCTTCG | GACAGTAGTCACATTGAACACYCGAAT |
| 5 | >R0129771713 | 1 | 29771713 | T | C | CAATTGCAGATTGCGTTGGATATCTTA | AATTGCAGATTGCGTTGGATATCTTG | GTGATTRCCTATAGACAGAATCACAGAAAT |
| 6 | >R0129975201 | 1 | 29975201 | A | G | TGTAACTGGATGTATGTAAAATCGAA | CTTGTAACTGGATGTATGTAAAATCGAG | TTTATGTGTCATTGTGRTGCATGTGAAAAT |
| 7 | >R0130035044 | 1 | 30035044 | C | T | TTAAGTGTATCTTGGAGACCACGAC | TTAAGTGTATCTTGGAGACCACGAT | GTGAAGGCCTTTTATGTCTGACA |
| 8 | >R0130038156 | 1 | 30038196 | A | G | CCATCTCTGCTGCTATATGGA | CCATCTCIGCTGCTATATGGG | CCATTACAAGACAGGGACCTT |
| 9 | >R0130038502 | 1 | 30038502 | A | T | TITAATACCTGTTTCCTGATAACGA | TTTAATACCTGTTICCTGATAACGT | GGCATGGAAGAGAGATGACC |
| 10 | >R0130038698 | 1 | 30038698 | T | C | GTGGCAGACTGGAGCATTCTGCGG | GTGGCAGACTGGAGCATTCTGCGA | AGGCTTCACAGATACGGTCA |
| 11 | >R0130038740 | 1 | 30038740 | A | T | TAGGTTATCTTGAAATTCCTCAGTTAA | TAGGTTATCTTGAAATTCCTCAGTTAT | CGCTCAAGGAACCAACCATC |
| 12 | >R0130038819 | 1 | 30038819 | T | C | ICTIGATGGCCTGCTIGGTGAT | TCTTGATGGCCTGCTTGGTGAG | TGCATGACAAGATTGATGAGC |
| 13 | >R0130039379 | 1 | 30039379 | C | A | TCCCTTTGGTTGCAAGTGCTTTTGAA | TCCCTTTGGTTGCAAGTGCTTTTGAC | ACATGGCTAGATATTCTGCTCCT |
| 14 | >R0130040473 | 1 | 30040473 | TTTG | T | CATTTTCCTCAGTAGTAAGCAAGAGTTT | TTCCTCAGTAGTAAGCAAGAGTTC | CATTGCTCCAAAACAGCGCTCTTCAA |
| 15 | >R0130057841 | 1 | 30057841 | T | G | GATGCTTGAATCTAGAAGTTGCCCA | ATGCTTGAATCTAGAAGTTGCCCG | ACATATCAGTAAAAAGTAGCACAATCTGTT |
| 16 | >R0130196202 | 1 | 30196202 | T | G | GTATAAGTTGTGCAGATTAAAATTATGTGCAA | ATAAGTTGTGCAGATTAAAATTATGTGCAG | GACCACTAACACGGAATTTTTGGAGATTA |
| 17 | >R0401849181 | 4 | 1849181 | T | G | CCTTCGTTGCTCGTTGTGAAATTCA | CTTCGTTGCTCGTTGTGAAATTCG | CAAGGAATCATCAACGATTCATGGTGAT |
| 18 | >R0601765761 | 6 | 1765761 | T | G | TCATCAGGAAGAACATCTGCAAGT | TCATCAGGAAGAACATCTGCAAGG | TTTCCAGCGCAACACCTTAG |
| 19 | >R0606752887 | 6 | 6752887 | CC | TT | AAGTACAAGGAGAGCTGGAGGGGGC | AAGTACAAGGAGAGCTGGAGGGGTT | ACCATTGGTACTTGGCCTTG |
| 20 | >R0721413833 | 7 | 21413833 | A | G | CAAACAAGAAAGGAGCGTTTTCAA | CTCAAACAAGAAAGGAGCGTTTTCAG | CCTACTRTTTGTGTTGGGCCAAAGT |
| 21 | >R0721599574 | 7 | 21599574 | A | G | GCCACCTGCTTTGACACCACT | GCCACCTGCTTTGACACCACC | GTGCTTACCAATTGGTCTACTTGATTGAT |
| 22 | >R1007303735 | 10 | 7303735 | A | T | CATACATTTACCTTTCTTTCTTAAATTATAAT | CATACATTTACCTTTCTTTCTTAAATTATAAA | CAAGCTACTAGCTAAATCTMCATCAAAGTT |
| 23 | >R1008803290 | 10 | 8803290 | T | C | ACACCYGAGGACATCATTAGTATGT | CACCYGAGGACATCATTAGTATGC | GGGCTAATTCCTCCTTGACTTARCATA |
| 24 | >R1008869299 | 10 | 8869299 | A | G | CCTTTTGTGCAATTTGAATAGTTATATTA | CTCCTTTTGTGCAATTTGAATAGTTATATTG | GATGACTTTACAAGAGAAAAGGGCATGAT |
| 25 | >R1008935229 | 10 | 8935229 | T | C | ATTAAAATCCTAAAATATCAGTATTGGAAATGA | AAAATCCTAAAATATCAGTATTGGAAATGG | CATAAGAGCATCACCAGTACCCCTA |
| 26 | >R1008984630 | 10 | 8984630 | A | G | GTTTATGAGTTTCACATAATTGGTCAGAAA | TATGAGTTTCACATAATTGGTCAGAAG | CTTGCTGTTTCTGTACCATGGCCTT |
| 27 | >R1009195457 | 10 | 9195457 | G | C | ATGTTATTATAACATAACTTTATCTAATATG | CTATGTTATTATAACATAACTTTATCTAATATC | GAGCTAAGGATATTACTGTTGTCRATAGTA |
| 28 | >R1009902249 | 10 | 9902249 | A | G | ACGAGGATTCACAGAWCAAAATATGTTTTT | CGAGGATTCACAGAWCAAAATATGTTTTC | GATATATGTAAGTTGTTCACTGGCTCTGT |
| 29 | >R1010217652 | 10 | 10217652 | A | T | CGCTACAAACATCACTAAGCAAAAATCA | CGCTACAAACATCACTAAGCAAAAATCT | TAGTTCGTGATGCAGTGCATATTCTGAAA |
| 30 | >R1011089916 | 10 | 11089916 | A | G | CACAACCAATTCATAAACTCCATACCA | ACAACCAATTCATAAACTCCATACCG | AAGCAAGATTTATCAAGCTACCAAGTTCTA |
| 31 | >R1011255488 | 10 | 11255488 | A | G | AGTGTATTCTCAAATGAACAAAATAATAACTAT | AGTGTATTCTCAAATGAACAAAATAATAACTAC | GTCAGGTGGTTTACATCTTGCAGCAT |
| 32 | >R1011259388 | 10 | 11259388 | T | C | TTATGGACGCCATCCACCATT | CTTTATGGACGCCATCCACCATC | ATGAGGAGATCATAGGAGCTCCATTATAT |
| 33 | >R1011274471 | 10 | 11274471 | A | T | GAACCAATCTTATCACCTAGCGCT | GAACCAATCTTATCACCTAGCGCA | GGGCCACCATCAGCCGCGAT |
| 34 | >R1222020155 | 12 | 22020155 | T | C | GTTCAGTGTTCAACATCAATCCCAA | GTTCAGTGTTCAACATCAATCCCAG | CTAGACTACTCCTTGACTAACTTGGWAA |
| 35 | >R1223010774 | 12 | 23010774 | T | G | GGCTCAGGAAGCGATCCTTCT | GGCTCAGGAAGCGATCCTTCG | CATGTTCTCCTGGGCMAAGTCCAA |

**Supplementary Table 7.** Genotyping data of the markers for CRE in the BC_1_F_2_ mapping population.

| **No.** | **Line** | **CRE ratio** | **R0129312181** | **R0129322283** | **R0129324976** | **R0129733026** | **R0129771713** | **R0129975201** | **R0130038156** | **R0130038502** | **R0130038819** | **R0130057841** | **R0130196202** | **R0401849181** | **R0601765761** | **R0606752887** | **R0721413833** | **R0721599574** | **R1007303735** | **R1008803290** | **R1008869299** | **R1008935229** | **R1008984630** | **R1009195457** | **R1009902249** | **R1010217652** | **R1011089916** | **R1011255488** | **R1011259388** | **R1011274471** | **R1222020155** | **R1223010774** |
| --- | --- | --- | --- | --- | --- | --- | --- | --- | --- | --- | --- | --- | --- | --- | --- | --- | --- | --- | --- | --- | --- | --- | --- | --- | --- | --- | --- | --- | --- | --- | --- | --- |
|  | #20A09 | 1.37 | C:C | C:C | A:A | T:T | C:C | G:G | A:A | A:A | C:C | T:T | G:G | G:G | G/G | TT/TT | G:G | G:G | T:T | C:C | A:A | C:C | G:G | G:G | G:G | A:A | A:A | A:A | T:T | A:A | T:T | G:G |
|  | NHN | 1.72 | T:T | T:T | G:G | C:C | T:T | A:A | G:G | T:T | T:T | G:G | T:T | T:T | T/T | GC/GC | A:A | A:A | A:A | T:T | G:G | T:T | A:A | C:C | A:A | T:T | G:G | G:G | C:C | T:T | C:C | T:T |
| 1 | 01_A06_50_03 | 1.42 | C:C | C:C | A:A | T:T | C:C | G:G | A:A | A:A | C:C | T:T | G:G | T:T | T/G | TT/TT | G:A | G:A | A:A | C:C | A:A | C:C | G:A | G:G | G:A | T:A | G:G | G:G | C:C | T:T | T:C | G:T |
| 2 | 01_A06_50_05 | 1.64 | T:T | T:T | G:G | C:C | T:T | A:A | G:G | T:T | T:T | G:G | T:T | T:T | G/G | TT/TT | G:A | G:A | A:T | C:T | G:A | C:T | G:A | G:C | G:A | T:A | G:G | G:G | C:C | T:T | C:C | T:T |
| 3 | 01_A06_50_06 | 1.64 | T:T | T:T | G:G | C:C | T:T | A:A | G:G | T:T | T:T | G:G | G:G | G:T | T/G | TT/TT | G:A | G:A | A:T | C:T | G:A | C:T | G:A | G:C | A:A | T:A | G:A | G:A | C:T | A:T | T:T | T:T |
| 4 | 01_A07_51_01 | 1.41 | C:C | C:C | A:A | T:T | C:C | G:G | G:A | A:T | C:T | T:T | G:G | T:T | T/G | GC/TT | G:A | G:A | A:T | C:T | G:A | C:T | G:A | G:C | A:A | T:A | G:A | G:A | C:T | A:T | T:C | G:T |
| 5 | 01_A07_51_02 | 1.58 | C:T | C:T | G:A | C:T | C:T | A:A | G:A | A:T | C:T | G:G | G:G | G:T | T/T | TT/TT | G:G | G:A | A:T | C:T | G:A | C:T | G:A | G:C | G:A | T:A | G:A | G:A | C:T | A:T | T:C | G:T |
| 6 | 01_A07_51_04 | 1.63 | T:T | T:T | G:G | C:C | T:T | A:A | G:G | T:T | T:T | G:G | T:T | T:T | T/G | TT/TT | G:A | G:A | T:T | C:C | A:A | C:C | G:G | G:G | A:A | A:A | A:A | A:A | T:T | A:A | T:C | T:T |
| 7 | 01_A07_51_05 | 1.59 | C:T | C:T | G:A | C:T | C:T | G:A | G:A | A:T | C:T | G:T | G:T | G:G | T/T | GC/TT | A:A | A:A | A:T | C:T | G:A | C:T | G:A | G:C | G:A | T:A | G:A | G:A | C:T | A:T | C:C | G:G |
| 8 | 01_A07_51_09 | 1.56 | C:T | C:T | G:A | C:T | C:T | G:A | G:A | A:T | C:T | G:T | G:T | T:T | T/T | GC/TT | G:A | G:A | A:T | C:T | G:A | C:T | G:A | G:C | A:A | T:A | G:A | G:A | C:T | A:T | T:C | G:T |
| 9 | 01_B04_34_04 | 1.75 | C:T | C:T | G:A | C:T | C:T | G:A | G:G | A:T | C:T | G:T | G:T | G:T | T/G | GC/TT | G:A | G:A | T:T | C:C | A:A | C:C | G:G | G:G | G:A | A:A | A:A | A:A | T:T | A:A | T:T | G:G |
| 10 | 01_B04_34_13 | 1.47 | C:T | C:T | G:A | C:T | C:T | G:A | G:A | A:T | C:T | G:T | G:T | T:T | T/T | GC/TT | G:G | A:A | T:T | C:C | A:A | C:C | G:G | G:G | G:A | A:A | A:A | A:A | T:T | A:A | T:T | G:G |
| 11 | 01_D11_34_01 | 1.39 | C:C | C:C | A:A | T:T | T:T | G:G | G:A | T:T | T:T | T:T | G:G | G:T | T/G | GC/GC | G:G | G:A | A:A | C:T | G:A | C:T | G:A | G:C | G:A | T:A | G:A | G:A | C:T | A:T | T:C | G:T |
| 12 | 01_D11_34_06 | 1.57 | C:T | C:T | G:A | C:T | T:T | G:G | G:G | A:T | C:T | G:T | G:T | G:G | T/G | GC/GC | A:A | G:G | A:T | C:T | G:A | C:T | G:A | G:C | G:A | T:A | G:A | G:A | C:T | A:T | T:C | G:T |
| 13 | 01_D11_34_10 | 1.39 | T:T | T:T | G:G | C:T | C:T | G:A | A:A | A:A | C:C | T:T | G:G | G:G | G/G | GC/TT | G:A | G:A | T:T | C:C | A:A | T:T | G:G | G:G | G:G | A:A | A:A | A:A | T:T | A:A | T:T | T:T |
| 14 | 01_E12_18_01 | 1.58 | T:T | T:T | G:G | C:C | T:T | A:A | G:G | T:T | T:T | G:G | T:T | T:T | T/G | GC/GC | G:A | G:A | T:T | C:C | A:A | T:T | G:G | G:G | G:G | A:A | A:A | A:A | T:T | A:A | C:C | G:G |
| 15 | 01_E12_18_07 | 1.65 | T:T | T:T | G:G | C:T | T:T | G:A | G:G | T:T | T:T | G:G | T:T | T:T | T/T | GC/GC | A:A | A:A | A:A | T:T | A:A | T:T | A:A | C:C | A:A | T:T | G:G | G:G | C:C | T:T | C:C | T:T |
| 16 | 01_E12_18_08 | 1.48 | T:T | T:T | G:G | C:C | T:T | A:A | A:A | T:T | T:T | G:G | T:T | T:T | T/T | GC/GC | G:A | G:G | A:A | T:T | G:G | T:T | A:A | C:C | A:A | T:A | G:G | G:G | C:C | T:T | C:C | T:T |
| 17 | 01_E12_18_10 | 1.52 | T:T | T:T | G:G | C:C | T:T | A:A | G:G | T:T | T:T | G:G | T:T | T:T | T/G | GC/GC | G:A | G:A | T:T | C:C | A:A | C:C | G:G | G:G | G:G | A:A | A:A | A:A | T:T | A:A | C:C | T:T |
| 18 | 01_F05_56_01 | 1.38 | C:T | C:T | G:A | T:T | C:T | G:A | A:A | A:A | C:C | G:T | G:T | G:G | T/G | GC/TT | G:A | G:A | T:T | C:C | A:A | C:C | G:G | G:G | G:G | A:A | A:A | A:A | T:T | A:A | T:T | G:G |
| 19 | 01_F05_56_04 | 1.73 | C:T | C:T | G:A | C:T | C:T | G:A | G:A | A:T | C:T | G:T | G:T | T:T | G/G | GC/TT | G:G | G:G | T:T | C:C | A:A | C:C | G:G | G:G | G:G | A:A | A:A | A:A | T:T | A:A | T:C | G:T |
| 20 | 01_F05_56_06 | 1.30 | C:T | C:T | G:A | C:T | C:T | G:A | A:A | A:T | C:T | G:T | G:T | T:T | T/G | GC/TT | G:A | G:A | T:T | C:C | A:A | C:C | G:G | G:G | G:G | A:A | A:A | A:A | T:T | A:A | C:C | T:T |
| 21 | 01_F05_56_07 | 1.60 | C:C | C:C | A:A | C:T | C:T | G:A | G:G | A:A | C:C | T:T | G:G | G:T | T/G | GC/TT | G:G | A:A | A:T | C:T | G:A | C:T | G:A | G:C | G:A | T:A | G:A | G:A | C:T | A:T | T:C | G:T |
| 22 | 01_F05_56_08 | 1.57 | C:C | C:C | A:A | T:T | C:C | G:G | A:A | A:A | C:C | T:T | G:G | G:T | T/T | GC/GC | G:A | G:G | T:T | C:C | A:A | C:C | G:G | G:G | G:G | A:A | A:A | A:A | T:T | A:A | T:T | G:G |
| 23 | 01_G10_53_04 | 1.59 | C:T | T:T | G:G | C:C | T:T | A:A | A:A | T:T | T:T | G:G | T:T | G:T | T/G | TT/TT | A:A | A:A | A:A | T:T | G:G | T:T | A:A | C:C | A:A | T:A | G:G | G:G | C:C | T:T | C:C | T:T |
| 24 | 01_G10_53_05 | 1.52 | C:T | C:T | G:A | C:T | C:T | G:A | G:A | A:T | C:T | G:T | G:T | G:T | G/G | GC/TT | G:A | G:A | A:A | C:T | G:A | C:T | G:A | G:C | G:A | T:A | G:G | G:A | C:T | A:T | C:C | G:T |
| 25 | 01_G10_53_06 | 1.58 | C:T | C:T | G:A | C:T | C:T | G:A | G:A | A:T | C:T | G:T | G:T | G:G | G/G | GC/TT | G:A | G:A | A:A | C:C | G:A | C:T | G:A | G:C | G:A | T:A | G:A | G:A | C:T | A:T | T:C | G:T |
| 26 | 01_G10_53_07 | 1.66 | T:T | T:T | G:G | C:C | T:T | A:A | G:G | T:T | T:T | G:G | T:T | G:G | G/G | TT/TT | A:A | A:A | A:A | T:T | G:G | T:T | A:A | C:C | A:A | T:A | G:G | G:G | C:C | T:T | C:C | T:T |
| 27 | 01_G10_53_08 | 1.47 | C:T | T:T | G:G | C:C | T:T | A:A | G:A | T:T | T:T | G:G | T:T | G:G | T/T | GC/GC | A:A | A:A | A:A | T:T | G:G | T:T | A:A | C:C | A:A | T:A | G:G | G:G | C:C | T:T | C:C | T:T |
| 28 | 01_G10_53_09 | 1.38 | C:C | C:C | A:A | T:T | C:C | G:G | G:A | T:T | T:T | T:T | G:G | G:T | T/G | TT/TT | G:G | A:A | A:T | C:T | G:A | C:T | G:A | G:C | G:A | T:A | G:G | G:G | C:T | A:T | T:C | G:T |
| 29 | 02_A02_49_01 | 1.48 | C:T | C:T | G:A | C:C | T:T | G:A | G:G | T:T | T:T | G:T | G:T | G:T | G/G | TT/TT | G:A | G:A | T:T | C:C | A:A | C:C | G:G | G:G | G:G | A:A | G:A | A:A | T:T | A:T | T:C | T:T |
| 30 | 02_A02_49_01 | 1.59 | T:T | T:T | G:G | C:C | T:T | A:A | G:G | T:T | T:T | G:G | T:T | G:T | G/G | GC/TT | G:A | A:A | T:T | C:C | A:A | C:C | G:G | G:G | G:G | A:A | A:A | A:A | T:T | A:A | C:C | T:T |
| 31 | 02_A02_49_03 | 1.50 | T:T | T:T | G:G | C:C | T:T | A:A | G:G | T:T | T:T | G:G | T:T | T:T | T/G | GC/TT | G:A | A:A | T:T | C:C | A:A | C:C | G:G | G:G | G:G | A:A | A:A | A:A | T:T | A:A | T:C | G:T |
| 32 | 02_A02_49_08 | 1.41 | T:T | T:T | G:G | C:C | T:T | A:A | G:G | T:T | T:T | G:G | T:T | G:T | G/G | GC/TT | G:G | A:A | A:T | C:T | G:A | C:T | G:A | G:C | G:A | T:A | G:A | G:A | C:T | A:T | C:C | T:T |
| 33 | 02_D03_45_04 | 1.42 | C:C | C:C | A:A | T:T | C:C | G:G | A:A | A:A | C:C | T:T | G:G | G:T | T/G | TT/TT | G:A | G:G | T:T | C:C | A:A | C:C | G:G | G:G | G:G | A:A | A:A | G:A | T:T | A:A | T:C | G:T |
| 34 | 02_D03_45_05 | 1.55 | C:C | C:C | A:A | T:T | C:C | G:G | A:A | A:A | C:C | T:T | G:G | G:T | T/T | TT/TT | G:A | A:A | A:T | C:T | G:A | C:T | G:A | G:C | G:A | T:A | G:A | G:A | C:T | A:T | T:C | G:T |
| 35 | 02_D07_44_01 | 1.43 | C:C | C:C | A:A | T:T | C:C | G:G | A:A | A:A | C:C | T:T | G:G | T:T | T/T | TT/TT | A:A | A:A | T:T | C:C | A:A | C:C | G:G | G:G | G:G | A:A | A:A | A:A | T:T | A:A | T:T | G:G |
| 36 | 02_D07_44_04 | 1.66 | T:T | T:T | G:G | C:C | T:T | A:A | G:G | T:T | T:T | G:G | T:T | T:T | T/T | GC/TT | A:A | A:A | A:A | C:T | G:G | T:T | A:A | C:C | A:A | T:T | G:G | G:G | C:C | T:T | C:C | T:T |
| 37 | 02_D07_44_08 | 1.51 | C:T | C:T | G:A | C:T | C:T | G:A | G:A | A:T | C:T | G:T | G:T | T:T | T/T | TT/TT | A:A | A:A | A:A | T:T | G:G | T:T | A:A | C:C | A:A | T:A | G:G | G:G | C:C | T:T | C:C | T:T |
| 38 | 02_D07_44_09 | 1.54 | C:T | C:T | G:A | C:T | C:T | G:A | G:A | A:T | C:T | G:T | G:T | T:T | T/T | GC/GC | A:A | A:A | T:T | C:C | A:A | C:C | G:G | G:G | G:G | T:A | G:A | G:A | C:T | A:T | C:C | T:T |
| 39 | 02_D07_44_10 | 1.48 | C:T | C:T | G:A | C:T | C:T | G:A | G:A | A:T | C:T | G:T | G:T | T:T | T/T | GC/TT | A:A | A:A | A:T | C:T | G:A | C:T | G:A | G:C | G:A | T:A | G:A | G:A | C:T | A:T | C:C | T:T |
| 40 | 02_F04_26_01 | 1.76 | T:T | T:T | G:G | C:C | T:T | A:A | G:G | T:T | T:T | G:T | T:T | G:T | T/G | GC/GC | G:A | G:A | A:T | C:T | G:G | T:T | A:A | C:C | A:A | T:A | G:G | G:G | C:T | T:T | T:C | G:T |
| 41 | 02_F04_26_02 | 1.53 | T:T | T:T | G:G | C:C | T:T | A:A | G:G | A:T | C:T | G:G | T:T | T:T | T/G | GC/GC | G:A | G:A | A:T | C:T | G:A | C:T | G:A | G:C | G:A | T:A | G:A | G:A | C:T | A:T | C:C | T:T |
| 42 | 02_F04_26_04 | 1.42 | T:T | T:T | G:G | C:C | T:T | A:A | G:G | T:T | T:T | G:G | T:T | T:T | T/G | GC/GC | G:A | A:A | A:T | C:T | G:A | C:T | G:A | G:C | G:A | T:A | G:A | G:A | C:T | A:T | C:C | T:T |
| 43 | 02_F04_26_05 | 1.72 | T:T | T:T | G:G | C:C | T:T | A:A | G:G | T:T | T:T | G:G | T:T | T:T | G/G | GC/GC | G:A | G:G | T:T | C:C | A:A | C:C | G:G | G:G | G:G | A:A | A:A | A:A | T:T | A:A | C:C | T:T |
| 44 | 02_F09_29_01 | 1.71 | T:T | T:T | G:G | C:C | T:T | A:A | G:G | T:T | T:T | G:G | T:T | T:T | T/G | GC/GC | G:A | A:A | A:T | C:T | G:A | C:T | G:A | G:C | G:A | T:A | G:A | G:A | C:T | A:T | T:C | G:T |
| 45 | 02_F09_29_02 | 1.44 | C:T | C:T | G:A | C:T | C:T | G:A | G:G | T:T | T:T | G:T | G:T | G:G | G/G | GC/TT | A:A | A:A | A:T | C:T | G:A | C:T | G:A | G:C | G:A | T:A | G:A | G:A | C:T | A:T | T:C | G:T |
| 46 | 02_F09_29_04 | 1.65 | T:T | T:T | G:G | C:C | T:T | A:A | G:G | T:T | T:T | G:G | T:T | G:G | T/G | GC/GC | G:A | G:A | A:A | T:T | G:G | T:T | A:A | C:C | A:A | T:A | G:G | G:G | C:C | T:T | T:C | G:T |
| 47 | 02_F09_29_09 | 1.61 | C:T | C:T | G:A | C:T | C:T | G:A | G:G | T:T | T:T | G:G | T:T | G:G | G/G | GC/GC | G:A | G:A | A:T | C:T | G:A | C:T | G:A | G:C | G:A | T:A | G:A | G:A | C:T | A:T | C:C | T:T |
| 48 | 02_G01_52_06 | 1.51 | T:T | T:T | G:A | C:T | C:T | G:A | G:A | A:T | C:T | G:T | T:T | T:T | G/G | TT/TT | A:A | A:A | A:T | C:T | G:A | C:T | G:A | G:C | G:A | T:T | G:G | G:G | C:C | T:T | C:C | T:T |
| 49 | 02_G01_52_07 | 1.47 | T:T | T:T | G:G | C:C | T:T | A:A | G:G | T:T | T:T | G:G | T:T | T:T | T/G | GC/TT | A:A | A:A | A:T | C:C | G:A | C:T | G:A | G:C | G:A | T:T | G:A | G:G | C:C | T:T | C:C | T:T |
| 50 | 02_G01_52_09 | 1.53 | C:C | C:C | A:A | T:T | C:C | G:G | A:A | A:A | C:C | T:T | G:G | T:T | T/G | TT/TT | A:A | A:A | A:T | C:T | G:A | C:T | G:A | G:C | G:A | T:T | G:G | G:G | C:C | T:T | C:C | T:T |
| 52 | 02_G01_52_10 | 1.52 | T:T | T:T | G:G | C:C | T:T | A:A | G:G | T:T | T:T | G:G | T:T | T:T | G/G | GC/TT | A:A | A:A | T:T | C:C | A:A | C:C | G:A | G:G | G:G | T:T | G:A | G:G | C:C | T:T | T:C | T:T |
| 51 | 02_G01_52_10 | 1.71 | T:T | T:T | G:G | C:C | T:T | A:A | G:A | A:T | C:T | G:G | T:T | T:T | T/G | GC/TT | A:A | A:A | T:T | C:C | A:A | C:C | G:G | G:G | G:G | T:T | G:G | G:G | C:C | T:T | C:C | T:T |
| 53 | 02_G08_29_01 | 1.51 | T:T | T:T | G:G | C:C | T:T | A:A | G:G | T:T | T:T | G:G | T:T | G:G | T/G | GC/GC | G:A | G:A | A:A | C:T | G:G | T:T | A:A | C:C | A:A | T:T | G:G | G:G | C:C | T:T | T:C | G:T |
| 54 | 02_G08_29_02 | 1.48 | T:T | T:T | G:G | C:C | T:T | A:A | G:G | A:T | C:T | G:G | T:T | G:G | T/G | GC/GC | G:A | G:A | A:A | T:T | G:G | T:T | A:A | C:C | A:A | T:T | G:G | G:G | C:C | T:T | T:T | G:G |
| 55 | 02_G08_29_03 | 1.33 | T:T | T:T | G:G | C:C | T:T | A:A | G:A | A:T | C:T | T:T | G:G | G:T | T/T | GC/TT | G:A | G:A | A:A | T:T | G:G | T:T | A:A | C:C | A:A | T:T | G:G | G:G | C:C | T:T | T:T | G:G |
| 56 | 02_G08_29_04 | 1.62 | T:T | T:T | G:G | C:C | T:T | A:A | G:G | A:T | C:T | G:G | T:T | G:T | T/G | GC/GC | G:A | G:A | A:A | C:C | G:G | T:T | A:A | C:C | A:A | T:T | G:G | G:G | C:C | T:T | T:C | G:T |
| 57 | 02_G08_29_05 | 1.38 | T:T | T:T | G:G | C:C | T:T | A:A | A:A | T:T | T:T | T:T | G:G | G:T | T/G | GC/TT | A:A | A:A | A:A | T:T | G:G | T:T | A:A | C:C | A:A | T:T | G:G | G:G | C:C | T:T | T:C | G:T |
| 58 | 02_G08_29_08 | 1.53 | T:T | T:T | G:G | C:C | T:T | A:A | G:G | T:T | T:T | G:G | T:T | G:T | T/G | GC/GC | G:A | A:A | A:A | T:T | G:G | T:T | A:A | C:C | A:A | T:T | G:G | G:G | C:C | T:T | T:T | G:G |
| 59 | 02_G08_29_09 | 1.63 | C:T | C:T | G:A | C:T | C:T | G:A | G:G | T:T | T:T | G:T | G:T | T:T | T/T | GC/GC | G:A | G:A | A:T | C:T | G:A | C:T | G:A | G:C | G:A | T:A | G:A | G:A | C:T | A:T | C:C | T:T |
| 60 | 02_G08_29_10 | 1.48 | T:T | T:T | G:G | C:C | T:T | A:A | G:G | T:T | T:T | G:G | T:T | G:G | T/G | GC/GC | G:A | G:A | A:A | T:T | G:G | T:T | A:A | C:C | A:A | T:T | G:G | G:G | C:C | T:T | C:C | T:T |
| 61 | 03_C06_58_04 | 1.51 | C:T | C:T | G:A | C:T | C:T | G:A | G:A | A:T | C:T | G:T | G:T | T:T | T/G | TT/TT | G:G | A:A | T:T | C:C | A:A | C:C | G:G | G:G | G:G | A:A | A:A | A:A | T:T | A:A | C:C | T:T |
| 62 | 03_C06_58_11 | 1.68 | C:T | C:T | G:A | C:T | C:T | G:A | G:A | A:T | C:T | G:T | G:T | T:T | T/G | TT/TT | G:A | G:G | A:T | C:T | G:A | C:T | G:A | G:C | G:A | T:A | G:A | G:A | C:T | A:T | C:C | G:T |
| 63 | 03_C06_58_13 | 1.22 | C:C | C:C | A:A | T:T | C:C | G:G | A:A | A:A | C:C | T:T | G:G | T:T | T/G | TT/TT | G:G | G:A | A:T | C:T | G:A | C:T | G:A | G:C | G:A | T:A | G:A | G:A | C:T | A:T | C:C | G:G |
| 64 | 03_D03_52_09 | 1.40 | C:T | C:T | G:A | C:T | C:T | G:A | G:A | A:T | C:T | G:T | G:T | T:T | G/G | TT/TT | G:G | A:A | A:A | C:T | G:G | T:T | A:A | C:C | A:A | T:T | G:G | G:G | C:C | T:T | C:C | T:T |
| 65 | 04_D08_45_01 | 1.47 | T:T | T:T | G:G | C:C | T:T | A:A | A:A | A:T | C:T | G:G | T:T | T:T | T/T | GC/GC | G:A | G:A | T:T | C:C | A:A | C:C | G:G | G:G | G:G | A:A | A:A | A:A | T:T | A:A | T:C | G:T |
| 66 | 04_D08_45_01 | 1.60 | T:T | T:T | G:G | C:C | T:T | A:A | G:G | T:T | T:T | G:G | T:T | T:T | T/T | GC/TT | G:A | G:A | T:T | C:C | A:A | C:C | G:G | G:G | G:G | A:A | A:A | A:A | T:T | A:A | T:C | G:T |
| 67 | 04_E03_35_01 | 1.48 | T:T | T:T | G:G | C:C | T:T | A:A | G:G | T:T | T:T | G:G | T:T | G:G | T/G | GC/GC | G:G | A:A | A:A | T:T | G:G | T:T | A:A | C:C | A:A | T:T | G:G | G:G | C:C | T:T | C:C | T:T |
| 68 | 04_E03_35_05 | 1.40 | C:C | C:C | A:A | T:T | C:C | G:G | A:A | T:T | T:T | T:T | G:G | T:T | G/G | GC/TT | G:A | A:A | A:A | T:T | G:G | T:T | A:A | C:C | A:A | T:T | G:G | G:G | C:C | T:T | C:C | T:T |
| 69 | 04_E03_35_07 | 1.57 | T:T | T:T | G:G | C:C | T:T | A:A | A:A | T:T | T:T | G:G | T:T | G:G | T/G | GC/TT | G:A | A:A | A:A | T:T | G:G | T:T | A:A | C:C | A:A | T:T | G:G | G:G | C:C | T:T | C:C | T:T |
| 70 | 08_A11_24_01 | 1.64 | T:T | T:T | G:G | C:C | T:T | A:A | G:A | A:T | C:T | G:G | T:T | G:G | G/G | GC/TT | G:G | A:A | A:A | T:T | G:G | T:T | A:A | C:C | A:A | T:T | G:G | G:G | C:C | T:T | C:C | T:T |
| 71 | 08_A11_24_02 | 1.32 | C:T | C:T | G:A | C:T | T:T | G:A | A:A | A:T | C:T | G:T | G:T | T:T | G/G | GC/TT | G:A | A:A | A:T | C:T | G:A | C:T | G:A | G:C | G:A | T:A | G:A | G:A | C:T | A:T | T:C | G:T |
| 72 | 08_A11_24_02 | 1.43 | C:T | C:T | G:A | C:T | C:T | G:A | G:A | A:T | C:T | G:T | G:T | T:T | T/T | GC/GC | G:A | A:A | T:T | C:C | A:A | C:C | G:G | G:G | G:G | A:A | A:A | A:A | T:T | A:A | T:C | G:T |
| 73 | 08_A11_24_03 | 1.38 | C:C | C:C | A:A | T:T | C:C | G:G | A:A | A:A | C:C | T:T | G:G | T:T | T/T | GC/GC | G:A | A:A | A:T | C:T | G:A | C:T | G:A | G:C | G:A | T:A | G:A | G:A | C:T | A:T | T:C | G:T |
| 74 | 08_A11_24_04 | 1.49 | C:T | C:T | G:A | C:T | C:T | G:A | G:A | A:T | C:T | G:T | G:T | T:T | T/G | GC/GC | G:A | A:A | A:T | C:T | G:A | C:T | G:A | G:C | G:A | T:A | G:A | G:A | C:T | A:T | T:T | G:G |
| 75 | 08_A11_24_05 | 1.41 | C:T | C:T | G:A | C:T | C:T | G:A | A:A | A:A | C:C | G:T | G:T | T:T | T/T | GC/GC | G:A | A:A | A:T | C:T | G:A | C:T | G:A | G:C | G:A | T:A | G:A | G:A | C:T | A:T | T:C | G:T |
| 76 | 08_A11_24_06 | 1.44 | C:T | C:T | G:A | C:T | C:T | G:A | G:A | A:T | C:T | G:T | G:T | T:T | G/G | GC/GC | A:A | A:A | A:T | C:T | G:A | C:T | G:A | G:C | G:A | T:A | G:A | G:A | C:T | A:T | T:C | G:T |
| 77 | 08_A11_24_07 | 1.43 | C:T | C:T | G:A | C:T | C:T | G:A | A:A | A:A | C:C | G:T | G:T | T:T | T/G | GC/TT | G:A | G:A | A:T | C:T | G:A | C:T | G:A | G:C | G:A | T:A | G:A | G:A | C:T | A:T | T:C | G:T |
| 78 | 08_A11_24_08 | 1.48 | C:T | C:T | G:A | C:T | C:T | G:A | G:A | A:T | C:T | G:T | G:T | T:T | T/G | GC/GC | G:A | A:A | A:T | C:T | G:A | C:T | G:A | G:C | G:A | T:A | G:A | A:A | T:T | A:A | T:C | G:T |
| 79 | 08_A11_24_09 | 1.53 | C:T | C:T | G:A | C:T | C:T | G:A | G:A | A:T | C:T | G:T | G:T | T:T | G/G | GC/GC | A:A | A:A | T:T | C:C | A:A | C:C | G:G | G:G | G:G | A:A | A:A | A:A | T:T | A:A | C:C | T:T |
| 80 | 08_C02_25_10 | 1.53 | C:T | C:T | G:A | C:T | C:T | G:A | G:A | A:T | C:T | G:T | G:T | G:T | T/G | GC/TT | G:A | G:G | A:A | C:T | G:A | C:T | G:A | G:C | G:A | T:A | G:A | G:G | C:T | A:T | T:C | G:G |
| 81 | 08_C02_27_10 | 1.57 | C:T | C:T | G:A | C:T | C:T | G:A | G:A | A:T | C:T | G:T | G:T | G:T | G/G | GC/TT | G:A | A:A | A:A | C:T | G:A | C:T | G:A | G:C | G:A | T:A | G:A | G:G | C:T | A:T | C:C | G:T |
| 82 | 08_C10_27_09 | 1.64 | T:T | T:T | G:G | C:C | T:T | A:A | G:G | T:T | T:T | G:G | T:T | G:T | G/G | GC/TT | G:G | A:A | A:A | C:T | G:A | C:T | G:A | G:C | G:A | T:A | G:A | G:G | C:T | A:T | C:C | G:G |
| 83 | 08_G08_29_01 | 1.61 | T:T | T:T | G:G | C:C | T:T | A:A | G:A | T:T | T:T | G:G | T:T | G:G | T/G | GC/GC | G:A | A:A | A:A | T:T | G:G | T:T | A:A | C:C | A:A | T:T | G:G | G:G | C:C | T:T | T:T | G:G |
| 84 | 09_A11_12_01 | 1.42 | C:C | C:C | A:A | T:T | C:C | G:G | A:A | A:T | C:T | T:T | G:G | T:T | T/T | GC/GC | G:G | G:A | A:T | C:T | G:A | C:T | G:A | G:C | G:A | T:A | G:A | G:A | C:T | A:A | C:C | T:T |
| 85 | 09_A11_12_02 | 1.48 | C:C | C:C | A:A | T:T | C:C | G:G | G:G | A:T | C:T | T:T | G:G | T:T | T/T | GC/TT | A:A | A:A | T:T | C:C | A:A | C:C | G:G | G:G | G:G | A:A | A:A | A:A | T:T | A:A | T:C | G:T |
| 86 | 09_A11_12_03 | 1.44 | C:C | C:C | A:A | T:T | C:C | G:G | G:G | A:T | C:T | T:T | G:G | T:T | T/T | GC/GC | G:A | G:A | T:T | C:C | A:A | C:C | G:G | G:G | G:G | A:A | A:A | A:A | T:T | A:A | T:C | G:T |
| 87 | 09_A11_12_04 | 1.41 | C:C | C:C | A:A | T:T | C:C | G:G | G:A | A:T | C:T | T:T | G:G | T:T | G/G | GC/TT | A:A | G:A | T:T | C:C | A:A | C:C | G:G | G:G | G:G | A:A | A:A | A:A | T:T | A:A | C:C | T:T |
| 88 | 09_A11_12_05 | 1.63 | T:T | T:T | G:G | C:C | T:T | A:A | G:G | T:T | T:T | G:G | T:T | T:T | G/G | GC/TT | G:G | A:A | A:A | T:T | G:G | T:T | A:A | C:C | A:A | T:T | G:G | G:G | C:C | T:T | C:C | T:T |
| 89 | 09_A11_12_06 | 1.53 | C:T | C:T | G:A | C:T | C:T | G:A | G:A | A:T | C:T | G:T | G:T | T:T | T/T | GC/GC | G:G | A:A | A:A | T:T | G:G | T:T | A:A | C:C | A:A | T:A | G:G | G:G | C:C | T:T | C:C | T:T |
| 90 | 09_A11_12_07 | 1.44 | C:T | C:T | G:A | C:T | C:T | G:A | G:G | T:T | T:T | G:T | G:T | T:T | G/G | GC/TT | G:A | G:G | A:T | C:T | G:A | C:T | G:A | G:C | G:A | T:A | G:A | G:A | C:T | A:T | T:C | G:T |
| 91 | 09_A11_12_08 | 1.42 | C:T | C:T | G:G | C:C | T:T | A:A | G:G | T:T | T:T | G:G | T:T | T:T | T/T | GC/GC | G:G | G:A | A:T | C:T | G:A | C:T | G:A | G:C | G:A | T:A | G:A | G:A | C:T | A:T | C:C | T:T |
| 92 | 09_A11_12_09 | 1.43 | C:C | C:C | A:A | T:T | C:C | G:G | A:A | A:A | C:C | T:T | G:G | T:T | T/T | GC/GC | G:A | G:G | A:T | C:T | G:A | C:T | G:A | G:C | G:A | T:A | G:A | G:A | C:T | A:T | C:C | T:T |
| 93 | 09_G04_39_03 | 1.54 | T:T | T:T | G:G | C:C | T:T | A:A | G:G | T:T | T:T | G:G | T:T | G:T | T/T | TT/TT | A:A | A:A | A:A | T:T | G:G | T:T | A:A | C:C | A:A | T:T | G:G | G:G | C:C | T:T | C:C | T:T |
| 94 | 09_G04_39_04 | 1.56 | T:T | T:T | G:G | C:C | T:T | A:A | G:G | T:T | T:T | G:G | T:T | G:T | T/T | GC/GC | A:A | A:A | A:T | C:T | G:A | C:T | G:A | G:C | G:A | T:A | G:A | G:A | C:T | A:T | C:C | T:T |
| 95 | 09_G04_39_05 | 1.49 | T:T | T:T | G:G | C:C | T:T | A:A | G:G | T:T | T:T | G:G | T:T | G:T | T/T | GC/TT | A:A | A:A | T:T | C:C | A:A | C:C | G:G | G:G | G:G | A:A | A:A | A:A | T:T | A:A | C:C | T:T |
| 96 | 09_H10_31_10 | 1.48 | C:T | C:T | G:A | C:T | C:T | G:A | G:A | A:T | C:T | G:T | G:T | G:T | T/T | GC/GC | G:A | G:A | A:T | C:T | G:A | C:C | G:A | G:C | G:A | T:A | G:A | G:A | C:T | A:T | C:C | T:T |
| 97 | 09_H10_31_10 | 1.57 | C:T | C:T | G:A | C:T | C:T | G:A | G:A | A:T | C:T | G:T | G:T | T:T | T/T | GC/GC | G:A | G:G | A:T | C:T | G:A | C:T | G:A | G:C | G:A | T:A | G:A | G:A | C:T | A:T | C:C | T:T |
| 98 | 09_H11_12_02 | 1.40 | C:T | C:T | G:A | C:T | C:T | G:A | G:A | A:T | C:T | G:T | G:T | T:T | T/T | GC/GC | G:A | G:A | A:T | C:C | G:A | C:T | G:A | G:C | G:A | T:A | G:A | G:A | C:T | A:T | C:C | T:T |
| 99 | 09_H11_12_05 | 1.41 | T:T | T:T | G:G | C:C | T:T | A:A | A:A | A:T | C:T | G:G | T:T | T:T | T/T | GC/GC | G:G | G:A | A:T | C:T | G:A | C:T | G:A | G:C | G:A | T:A | G:A | G:A | C:T | A:T | C:C | T:T |
| 100 | 09_H11_12_06 | 1.55 | C:T | C:T | G:A | C:T | C:T | G:A | G:A | T:T | C:T | G:T | G:T | T:T | T/T | GC/GC | G:A | A:A | A:T | C:T | G:A | C:T | G:A | G:C | G:A | T:A | G:A | G:A | C:T | A:T | C:C | T:T |
| 101 | 09_H11_12_07 | 1.41 | C:T | C:T | G:A | C:T | C:T | G:A | G:A | A:T | C:T | G:T | G:T | T:T | T/T | GC/GC | G:A | A:A | T:T | C:C | A:A | C:C | G:G | G:G | G:G | A:A | A:A | A:A | T:T | A:A | C:C | T:T |
| 102 | 09_H11_12_10 | 1.47 | C:T | C:T | G:A | C:T | C:T | G:A | A:A | A:A | C:C | G:T | G:T | G:G | T/T | GC/GC | G:G | A:A | T:T | C:C | A:A | C:C | G:G | G:G | G:G | A:A | A:A | A:A | T:T | A:A | T:C | G:T |
| 103 | 09_H11_12_11 | 1.65 | T:T | T:T | G:G | C:C | T:T | A:A | G:G | T:T | T:T | G:G | T:T | T:T | T/T | GC/GC | G:A | A:A | A:A | C:T | G:G | T:T | A:A | C:C | A:A | T:T | G:G | G:G | C:C | T:T | T:C | G:T |
| 104 | 09_H11_12_13 | 1.48 | C:T | C:T | G:A | C:C | C:T | G:A | A:A | A:T | C:T | G:T | G:T | T:T | T/T | GC/GC | G:A | G:A | A:T | C:T | G:A | C:T | G:A | G:C | G:A | T:A | G:A | G:A | C:T | A:T | T:C | G:T |
| 105 | 09_H11_12_17 | 1.38 | C:C | C:T | A:A | T:T | C:C | G:G | G:A | A:T | C:T | T:T | G:G | T:T | T/T | GC/GC | G:A | G:A | T:T | C:C | A:A | C:C | G:G | G:G | G:G | A:A | A:A | A:A | T:T | A:A | T:T | G:G |

**Supplementary Table 8.** CRE ratios of the 31 germplasm lines.

| **No.** | **name** | **L0 (mm)** | | | | | | | | | | **AVG** | **L1 (mm)** | | | | | | | | | | **AVG** | **Ratio (L1-L0)** |
| --- | --- | --- | --- | --- | --- | --- | --- | --- | --- | --- | --- | --- | --- | --- | --- | --- | --- | --- | --- | --- | --- | --- | --- | --- |
|  |  | **S1** | **S2** | **S3** | **S4** | **S5** | **S6** | **S7** | **S8** | **S9** | **S10** |  | **S1** | **S2** | **S3** | **S4** | **S5** | **S6** | **S7** | **S8** | **S9** | **S10** |  |  |
| 1 | Paw San Hmwe | 5.47 | 5.14 | 5.47 | 5.59 | 5.14 | 5.38 | 5.39 | 5.13 | 5.63 | 4.88 | 5.32 | 11.01 | 10.93 | 11.23 | 10.83 | 10.42 | 10.89 | 11.29 | 9.34 | 11.63 | 11.05 | 10.86 | 2.04 |
| 2 | PB1885_153 | 9.54 | 9.82 | 9.10 | 9.25 | 9.69 | 9.56 | 9.63 | 9.52 | 9.51 | 9.66 | 9.53 | 17.43 | 17.92 | 16.38 | 19.20 | 19.98 | 18.30 | 18.16 | 18.07 | 17.39 | 19.44 | 18.23 | 1.91 |
| 3 | PB1885_154 | 9.33 | 9.38 | 8.64 | 8.98 | 9.07 | 9.16 | 9.20 | 8.86 | 9.16 | 9.58 | 9.14 | 17.74 | 17.26 | 17.04 | 16.83 | 18.23 | 17.15 | 16.88 | 17.58 | 17.34 | 18.11 | 17.42 | 1.91 |
| 4 | Pusa Basmati_1121 | 8.43 | 8.79 | 8.39 | 8.52 | 9.71 | 8.41 | 8.54 | 8.45 | 8.35 | 8.39 | 8.60 | 18.16 | 17.62 | 15.74 | 17.75 | 19.32 | 18.55 | 16.80 | 18.18 | 16.68 | 16.72 | 17.55 | 2.04 |
| 5 | PB1121_149 | 8.99 | 8.73 | 9.33 | 9.92 | 9.37 | 9.04 | 9.05 | 9.01 | 9.60 | 9.43 | 9.25 | 17.48 | 17.95 | 19.26 | 18.78 | 17.29 | 20.26 | 18.19 | 18.72 | 17.79 | 17.52 | 18.32 | 1.98 |
| 6 | PB1121_150 | 8.92 | 8.65 | 8.14 | 8.35 | 8.95 | 9.07 | 9.03 | 8.52 | 8.95 | 9.33 | 8.79 | 17.67 | 17.55 | 18.13 | 18.02 | 18.69 | 18.09 | 20.03 | 18.22 | 17.28 | 17.47 | 18.11 | 2.06 |
| 7 | PB1401_146 | 8.41 | 8.57 | 8.33 | 8.70 | 9.08 | 8.61 | 7.94 | 8.48 | 8.60 | 8.64 | 8.53 | 17.07 | 17.80 | 18.77 | 18.26 | 19.06 | 18.35 | 18.98 | 18.19 | 19.04 | 18.97 | 18.45 | 2.16 |
| 8 | PB1401_145 | 8.28 | 8.13 | 8.89 | 8.28 | 7.95 | 8.12 | 7.92 | 8.29 | 8.19 | 8.65 | 8.27 | 16.71 | 14.83 | 16.17 | 16.01 | 15.90 | 15.78 | 16.03 | 16.95 | 16.39 | 17.17 | 16.19 | 1.96 |
| 9 | Pusa Basmati_1718 | 8.83 | 9.15 | 8.98 | 9.10 | 8.87 | 8.56 | 8.20 | 8.65 | 8.94 | 8.99 | 8.83 | 16.94 | 18.79 | 18.55 | 15.53 | 18.56 | 19.01 | 18.46 | 19.02 | 17.73 | 18.75 | 18.13 | 2.05 |
| 10 | PB1718_155 | 8.90 | 8.82 | 9.25 | 8.24 | 8.88 | 9.11 | 9.54 | 8.84 | 8.96 | 9.17 | 8.97 | 18.39 | 18.22 | 18.52 | 18.82 | 17.29 | 19.51 | 18.98 | 16.70 | 16.88 | 19.55 | 18.28 | 2.04 |
| 11 | PB1718_156 | 8.79 | 8.56 | 8.43 | 9.92 | 9.44 | 8.74 | 9.49 | 9.25 | 9.49 | 9.33 | 9.14 | 18.20 | 16.42 | 16.64 | 18.12 | 17.56 | 16.53 | 17.81 | 18.39 | 19.39 | 18.04 | 17.71 | 1.94 |
| 12 | PB1509_147 | 9.58 | 9.16 | 9.12 | 9.75 | 9.62 | 9.18 | 9.20 | 9.20 | 9.49 | 9.57 | 9.39 | 17.50 | 18.42 | 15.52 | 16.19 | 16.59 | 16.53 | 17.88 | 18.58 | 17.19 | 17.85 | 17.22 | 1.84 |
| 13 | PB1509_148 | 8.92 | 8.80 | 9.32 | 8.82 | 9.14 | 9.75 | 9.26 | 9.24 | 8.80 | 8.12 | 9.02 | 16.70 | 16.43 | 17.51 | 17.18 | 15.73 | 17.59 | 15.76 | 16.94 | 14.39 | 13.38 | 16.16 | 1.79 |
| 14 | PB1692_152 | 9.64 | 9.16 | 9.75 | 9.32 | 8.56 | 8.39 | 9.04 | 8.60 | 9.16 | 9.13 | 9.07 | 16.74 | 17.46 | 18.86 | 16.80 | 16.43 | 16.18 | 16.08 | 16.02 | 17.29 | 16.36 | 16.82 | 1.85 |
| 15 | NHN | 8.56 | 7.95 | 8.69 | 8.31 | 8.12 | 7.74 | 8.56 | 8.48 | 8.10 | 8.44 | 8.29 | 14.69 | 14.76 | 14.26 | 14.32 | 14.54 | 13.40 | 14.54 | 14.35 | 13.56 | 14.25 | 14.27 | 1.72 |
| 16 | PB1692_151 | 9.54 | 9.72 | 9.82 | 9.08 | 9.18 | 9.31 | 9.93 | 8.55 | 9.11 | 9.24 | 9.35 | 16.50 | 18.47 | 17.30 | 17.33 | 16.63 | 17.28 | 19.57 | 15.25 | 16.98 | 15.86 | 17.12 | 1.83 |
| 17 | Niaw Luem Pua | 7.37 | 8.18 | 7.21 | 7.45 | 7.56 | 7.74 | 8.18 | 7.38 | 7.89 | 7.80 | 7.67 | 13.01 | 12.79 | 13.30 | 12.29 | 12.19 | 14.57 | 12.79 | 12.71 | 12.89 | 13.60 | 13.01 | 1.70 |
| 18 | Niaw Dam Cham Mai Pai_L | 6.62 | 6.79 | 6.10 | 7.05 | 6.08 | 6.00 | 5.88 | 6.41 | 5.94 | 5.96 | 6.28 | 10.35 | 11.54 | 10.64 | 10.68 | 10.53 | 11.60 | 10.00 | 11.19 | 12.29 | 12.14 | 11.09 | 1.77 |
| 19 | Niaw Dam Cham Mai Pai_W | 6.42 | 5.89 | 5.65 | 5.99 | 6.24 | 6.22 | 6.22 | 5.54 | 5.94 | 5.63 | 5.97 | 9.81 | 9.13 | 9.16 | 9.18 | 9.83 | 9.58 | 9.85 | 8.67 | 8.93 | 9.33 | 9.35 | 1.56 |
| 20 | Jao Khao Chiang Mai | 7.14 | 7.56 | 7.16 | 6.96 | 6.47 | 7.29 | 7.18 | 6.86 | 6.87 | 7.15 | 7.06 | 11.93 | 11.12 | 11.42 | 10.45 | 11.05 | 10.35 | 10.63 | 11.33 | 11.40 | 9.62 | 10.93 | 1.55 |
| 21 | I Thi | 3.50 | 3.45 | 3.32 | 3.38 | 3.45 | 3.43 | 3.61 | 3.55 | 3.65 | 3.53 | 3.49 | 5.10 | 5.19 | 5.53 | 5.20 | 5.26 | 5.35 | 5.31 | 5.44 | 5.51 | 5.34 | 5.32 | 1.53 |
| 22 | Khao Niaw Dam | 6.55 | 6.72 | 7.35 | 6.81 | 7.34 | 7.15 | 7.23 | 6.87 | 6.32 | 6.95 | 6.93 | 8.42 | 9.00 | 9.74 | 8.48 | 10.60 | 9.54 | 9.46 | 9.60 | 7.95 | 9.17 | 9.19 | 1.33 |
| 23 | Niaw Dam Maw 37 | 7.12 | 6.99 | 6.87 | 7.29 | 6.65 | 7.23 | 6.95 | 7.12 | 6.38 | 7.47 | 7.01 | 9.50 | 9.75 | 9.17 | 9.84 | 9.52 | 9.39 | 9.94 | 9.62 | 8.90 | 10.18 | 9.58 | 1.37 |
| 24 | Hom Bao Rai | 7.48 | 7.08 | 7.56 | 7.65 | 7.49 | 6.89 | 7.48 | 7.40 | 7.48 | 7.16 | 7.37 | 11.03 | 11.58 | 10.80 | 11.57 | 11.62 | 9.71 | 11.19 | 12.55 | 12.25 | 11.47 | 11.38 | 1.54 |
| 25 | Num Roo | 6.88 | 7.04 | 7.09 | 6.96 | 6.95 | 6.87 | 7.04 | 6.88 | 7.14 | 7.21 | 7.00 | 10.14 | 9.62 | 10.00 | 9.78 | 10.15 | 11.49 | 10.61 | 9.76 | 10.14 | 10.42 | 10.21 | 1.46 |
| 26 | Khao Kai Noi Leuang | 4.96 | 4.63 | 4.54 | 4.55 | 4.20 | 4.54 | 4.71 | 4.83 | 4.54 | 4.83 | 4.63 | 6.89 | 7.34 | 7.18 | 7.30 | 6.32 | 6.98 | 6.77 | 7.15 | 7.65 | 6.81 | 7.04 | 1.52 |
| 27 | Dok Kha 50 | 7.72 | 6.94 | 7.32 | 7.30 | 6.86 | 7.13 | 6.87 | 6.97 | 7.05 | 7.06 | 7.12 | 11.37 | 10.35 | 11.12 | 11.92 | 11.82 | 11.10 | 10.84 | 11.25 | 11.29 | 11.31 | 11.24 | 1.58 |
| 28 | DH103 | 7.40 | 7.06 | 6.98 | 7.83 | 7.24 | 6.90 | 7.65 | 7.23 | 7.32 | 7.15 | 7.28 | 12.17 | 11.58 | 11.22 | 11.11 | 11.98 | 11.68 | 11.37 | 11.34 | 11.54 | 11.55 | 11.55 | 1.59 |
| 29 | Med Fay 62 | 7.22 | 7.25 | 6.67 | 7.51 | 7.50 | 7.25 | 7.30 | 7.00 | 7.18 | 7.00 | 7.19 | 11.32 | 11.24 | 10.48 | 12.01 | 11.85 | 10.82 | 11.80 | 10.91 | 11.19 | 12.19 | 11.38 | 1.58 |
| 30 | Pink+4 (#20A09) | 7.39 | 7.15 | 7.41 | 7.61 | 7.38 | 7.20 | 7.52 | 7.40 | 7.04 | 7.28 | 7.34 | 10.30 | 9.64 | 10.05 | 9.92 | 10.48 | 10.26 | 9.92 | 10.08 | 10.30 | 10.10 | 10.10 | 1.38 |
| 31 | PTT1 | 7.40 | 6.73 | 7.40 | 7.35 | 6.99 | 7.57 | 7.57 | 7.42 | 7.23 | 7.65 | 7.33 | 10.42 | 9.78 | 10.55 | 10.74 | 9.66 | 10.25 | 10.92 | 10.42 | 10.09 | 11.53 | 10.44 | 1.42 |

## 1.3 Supplementary Figures

**Supplementary Figure 1.** Selection of individuals from the phenotypic extremes of the BC_1_F_2_ derived F_3_ seed population for bulked DNA construction used in QTL-seq analysis. Two contrasting DNA bulks were generated, consisting of 17 high-CRE lines and 15 low-CRE lines, representing the upper and lower tails of the cooked rice elongation (CRE) distribution.
**Supplementary Figure 2.** A highly significant ∆(SNP-index) region was identified on chromosome 4 between 1.76 and 1.85 Mb.

**Supplementary Figure 3.** A highly significant ∆(SNP-index) region was identified on chromosome 7 between 21.4 and 21.8 Mb.

**Supplementary Figure 4.** A highly significant ∆(SNP-index) region was identified on chromosome 10 between 7.3-9.8 and 10.2-11.3 Mb.

**Supplementary Figure 5.** A highly significant ∆(SNP-index) region was identified on chromosome 12 between 21.7 and 25.8 Mb.

**Supplementary Figure 6.** Screening CRE phenotype of 31 rice germplasm

**Supplementary Figure 6.** Screening CRE phenotype of 31 rice germplasm (continue)
